# Multi-modular structure of the gene regulatory network for specification and commitment of murine T cells

**DOI:** 10.3389/fimmu.2023.1108368

**Published:** 2023-01-31

**Authors:** Boyoung Shin, Ellen V. Rothenberg

**Affiliations:** Division of Biology and Biological Engineering, California Institute of Technology, Pasadena, CA, United States

**Keywords:** gene regulatory network, early T cell development, transcription factors, gene expression program, cell fate decision, thymus, epigenetic control, multipotency

## Abstract

T cells develop from multipotent progenitors by a gradual process dependent on intrathymic Notch signaling and coupled with extensive proliferation. The stages leading them to T-cell lineage commitment are well characterized by single-cell and bulk RNA analyses of sorted populations and by direct measurements of precursor-product relationships. This process depends not only on Notch signaling but also on multiple transcription factors, some associated with stemness and multipotency, some with alternative lineages, and others associated with T-cell fate. These factors interact in opposing or semi-independent T cell gene regulatory network (GRN) subcircuits that are increasingly well defined. A newly comprehensive picture of this network has emerged. Importantly, because key factors in the GRN can bind to markedly different genomic sites at one stage than they do at other stages, the genes they significantly regulate are also stage-specific. Global transcriptome analyses of perturbations have revealed an underlying modular structure to the T-cell commitment GRN, separating decisions to lose “stem-ness” from decisions to block alternative fates. Finally, the updated network sheds light on the intimate relationship between the T-cell program, which depends on the thymus, and the innate lymphoid cell (ILC) program, which does not.

## 1 Introduction

Conventional T cells provide lifelong protection against infection and cancer by recognizing their cognate antigens and mediating effector functions. To ensure that the host can exert various immune responses in a context-specific manner, T cells have extensive diversity in their sub-lineages. The unique properties of T cells stem from the intersections of functional similarities with different types of immune cells (1, 2). Both T and B cells utilize antigen receptors whose diversities are achieved by DNA recombination and selection mechanisms. Distinct from B cells though, T cells can differentiate to various subsets showing functional parallelism with distinct types of helper-like innate lymphoid cells (ILCs) and natural killer (NK) cells, which lack recombined antigen receptors but produce effector cytokines and/or perform cytolytic functions in response to environmental signals. In addition, T cells possess proliferative potential and generate self-renewing long-lived populations, which are features shared with the multipotent hematopoietic progenitor cells and stem cells, respectively.

A recent global comparison of a wide range of hematopoietic cell types found that lineage-specific transcription factor motif “signatures” distinguished the active chromatin patterns for nearly every major analyzed hematopoietic lineage, suggesting the impacts of distinct lineage-determining transcription factors, but that T-lineage cells notably failed to show any cell type-specific “signature” (3). How, then, is the T-cell identity established and robustly maintained, despite functions broadly overlapping with those of other immune cells? We propose that this distinctive positioning as T cells can be supported by combinatorial actions of transcription factors, instead of relying on a lineage-specific “master regulator”. T cells utilize many transcription factors that are commonly employed by other types of hematopoietic cells for their own respective lineage specification and function. However, by precisely regulating combinatorial actions of these transcription factors over time under the influence of Notch signaling, and possibly also using epigenetic chromatin changes to inhibit reversibility, intrathymic hematopoietic precursors launch and lock down the specific T-lineage program. In this analytical review, we bring together recent evidence that assembles a newly clear picture of how this comes about.

## 2 Gene regulatory network models as explanations of developmental pathways

### 2.1 Gene regulatory networks in development

Developmental progression to generate a specialized cell type requires continuous, ordered changes in gene expression (4, 5). As a regulatory program, development must thus be distinct both from the stable epigenetic mechanisms that maintain a mature cell type’s identity (e.g., superenhancers) and from random transcriptional “noise”, both of which have been much studied in cell lines (6, 7). While environmental signals are often essential to trigger and support development, the unidirectional regulatory cascade that emerges depends on the transcription factors that are present in the cell receiving the signals and their impacts on other transcription factors and cellular chromatin states. The genes encoding transcription factors and signaling components collectively determine the regulatory state of the cell, and then the genes they control (downstream genes) encoding effector molecules determine the functional identity of a cell.

All these genes, whether they encode signaling receptors, transcription factors, or effector proteins, are controlled by *cis*-regulatory modules encoded in the genomic DNA, such as enhancers, silencers, and insulators, largely *via* interactions with *trans*-acting sequence-specific transcription factors (8, 9). Specifically activated long noncoding RNAs (lncRNAs) can contribute to chromatin states as well, although their actions are still only characterized in a few cases (10–13). Therefore, the important components driving development, the genes and their regulatory modules, transfer information in a directional manner. For example, transcription factor “X” binds to the regulatory elements of target genes *Y* and *Z*, which in turn induces expression of other regulatory factors, “Y” and “Z”. Importantly, the activities of these *cis*-regulatory elements are never driven by single transcription factors alone, but rather by combinations of factors, even though one or another factor can be rate-limiting in a particular experimental situation (14–20). As a result, gene regulation cascades are not explained by a linear pathway but by a hierarchical network (21, 22). These features of developmental regulatory networks can be effectively captured by using topological network models, in which the functional interactions are represented as inputs and outputs. While there are some caveats about the interpretation of these networks for hematopoietic development (23, 24), topological models are indispensable for compiling evidence to explain cell state differences in terms of gene regulation mechanisms.

In this review, we will focus on the gene regulatory programs utilized in the early stages of thymic T-cell development, in which multipotent progenitor cells undergo definitive T-lineage commitment and establish T-cell identity. Some gene regulatory network (GRN) circuits have been shown to promote cell type stability, as in the pluripotency state of ES cells, while others have been shown to drive ultra-rapid, deterministic cascades of change, as in the early Drosophila embryo (25, 26). As shown below, early T cell development falls between these models. It includes both regulatory subcircuits that resist change and regulatory connections that enforce change; the stochastic balance between these network subcircuits is likely to underlie the distinctively asynchronous kinetics of T cell program entry (27, 28). We review the major regulatory genes that are involved in different sub-programs to establish T-cell fate, resolve coherent but distinct program modules that need to be deployed, and propose an updated gene regulatory network (GRN) model.

### 2.2 Technical requirements and challenges for accurate GRN construction

There are significant challenges and caveats of experimental strategies that are utilized to understand developmental GRNs. As transcription factors need to bind to specific sequences in the DNA in order to work, genome-wide transcription factor occupancy data should in principle help to map where the “direct” interactions of the putative regulatory factor occur. In the past fifteen years it has become relatively easy to map transcription factor binding across the genome by ChIP-seq (or CUT&RUN, or CUT&Tag) (29–31). Potential transcription factor inputs to active regulatory elements can also be mapped in the DNA even without direct evidence of transcription factor binding, based on the enrichment of their motifs in accessible chromatin in a given cell type (32–34). Mapping open chromatin by DNase-seq or ATAC-seq and using the cell type-specific enrichment of motifs predicted to be bound by given transcription factors (35–37) in the open chromatin has become a powerful way to predict which transcription factors may be important in that cell type (38–44). Even without any evidence that a particular target gene actually responds to the presence of the transcription factor itself, such predictive analysis can be a valuable step toward perceiving network relationships at a global level (39, 45). Adding measured evidence for actual transcription factor occupancy at sites around key genes, when possible, enables specific network predictions to be made (46, 47).

However, actually confronting binding-based or motif-based predictions with empirical tests of individual target gene responses to transcription factor activity perturbations has given a more complex picture. Binding should not be overinterpreted. Transcription factor-DNA interaction sites in a cell type are often much more numerous (order of 10^4^ sites) than the number of genes that change expression in response to the loss or gain of a given transcription factor in that cell type (order of 10^2^ genes) (48–51). This indicates that a given transcription factor’s occupancy is often dispensable for regulation of most of the genes that it binds. Even when the transcription factor may be required for the existence of the cell type, its binding to the promoter of a particular gene in that cell type can be functionally irrelevant for that gene. It is thus challenging to predict which binding sites are actually functional, or what mode of actions they mediate (activation vs. inhibition), solely based on the transcription factor occupancy pattern. In addition, it is not simple to assign a binding site to potential target genes, especially if the binding site is surrounded with multiple genes or when the binding site is distant from a promoter. Developmentally important enhancers of key genes in the T-cell gene regulatory network can be hundreds of kilobases (kb) away from the promoters (e.g. *Bcl11b*, *Ets1, Gata3, Id2;* also *Myc*) (52–59). Disrupting individual regulatory elements by deletion or a mutation of a specific motif sequences can reveal the enhancer-target gene link, but functional redundancy of regulatory elements can lead to underestimation (60–62).

For identification of the gene network linkages described here, therefore, we have required evidence from functional tests that acutely perturbed the levels of transcription factor proteins in a specific developmental context. This has been achieved by germline/conditional deletion using a Cre-*loxP* or CRISPR-Cas9 system, knocking down using RNA interference (RNAi), repression using CRISPR interference (CRISPRi), or acute overexpression/ectopic activation of the target gene, each of which was then followed by *in vivo* and/or *in vitro* phenotype scoring at the cellular, transcriptomic, and epigenomic levels. Furthermore, we have given greater weight to results from experiments where the role of a transcription factor was tested in a precise, relevant T-cell developmental context and time window. This was important because recent results of stage-specific perturbation tests have shown that the same transcription factor’s function may change or disappear entirely in a different context, or even at a different stage within the same lineage (49, 51, 63–68). We have sought to apply consistent statistical criteria to these expression differences and to emphasize relationships that have been independently confirmed.

Precise controlling of perturbation timing can be experimentally challenging, especially in the context of a developmental process, as the cells are constantly progressing forward to the next stage. Inadequately defined perturbation time-windows could lead cells to developmental deviation toward irrelevant fates prior to reaching the stages of interest, or allow cells to activate compensatory mechanisms. Wide perturbation time windows could also span states in which the same transcription factor plays different roles, which could complicate data interpretation. Also, although stage-specific perturbation approaches can capture the functional consequences of the perturbation effectively, it is difficult to dissect whether the phenotype is resulting from direct effect, indirect effect *via* gene network, or indirect effect *via* differential population survival. As noted above, simply detecting transcription factor binding in the vicinity of a possible target gene is not enough to prove a direct functional relationship.

These limitations and challenges are not completely avoided within the data sources that we have analyzed to construct the update of the early T-development GRN. However, support for specific sites through which a transcription factor could exert direct control of a target gene can be gleaned from the recent increase in available genome-wide transcription factor binding data together with measurements of local chromatin states such as chromatin accessibility, 3D chromatin structure, and changes in histone marks, when these data are coupled with analyses of transcription factor perturbation effects.

### 2.3 Construction of an updated T-cell specification GRN model

The current gene network model we present differs from previous versions (27, 69–74) in several ways. In particular, initial models for early T-cell developmental GRNs were based primarily on candidate gene measurements, due to technical considerations. Targeted assay systems such as qPCR were used to examine perturbation effects on sets of only 100-150 genes out of 10,000 expressed transcripts, focused mainly on high sensitivity monitoring of transcription factor coding genes. It is now routine to use RNA-seq to measure the entire transcriptome quantitatively in an unbiased manner with low numbers of input cells, both at the bulk population level and at the single-cell level. This reveals whole batteries of genes coregulated by a given transcription factor perturbation in the specific context, which help to identify the changes in developmental status that have been induced. Genome-wide transcriptome data processing pipelines also standardize accepted statistical criteria. Where available, single-cell transcriptome analyses are also useful to separate perturbation effects on cells within a lineage from perturbation effects on population balances between the lineage of interest and contaminants.

Another change has been the advent of better technology for acute loss of function as well as acute gain of function of transcription factor genes within a well-defined developmental time window. Whereas Cre excision required a separate mouse strain to be developed for each gene to be targeted, using Cas9-transgenic mice (75) as cell sources for *in vitro* T-cell differentiation has made it possible to use guide RNAs (gRNAs) targeting one or several genes anywhere in the genome to be introduced, to disrupt genes efficiently at any stage desired in the same genetic background. Previous analyses of transcription factor-target linkages in early T cell development have often depended on gain of function or ectopic expression experiments because these techniques change transcription factor levels acutely in a specific cell type with even faster kinetics. However, multiple recent examples have shown that transcription factor impacts on a network can differ markedly depending on the level of expression of the transcription factor protein (GATA3, PU.1, Runx), and levels may be hard to control in gain of function. While loss-of-function experiments can pose other problems (asynchronous, sometimes slow loss of targeted protein; potential viability losses in the affected cells; potential masking by redundancy), they measure effects of factors at their normal levels of expression. Thus, gain of function data have needed to be re-evaluated in light of corresponding loss of function data, and the manipulated levels of factor protein have needed to be compared to physiological levels.

To construct the network models shown below, we have compiled data from several sources where developmentally well-defined gain or loss of function perturbations were carried out, with all significantly responding genes from each perturbation study tabulated in **Table S1**. The studies used are described in **Box 1**. **Table S1** also compiles evidence of local binding by the transcription factor of interest around each functional target gene, wherever this evidence was available. Note that different perturbation experiments used as sources focused on different developmental time windows and were more or less sensitive to loss or gain of a given factor’s activity, depending on the normal expression baseline at that timepoint. Where there was variation between different controls or inadequate expression of a gene within the time window tested, even repeatedly observed effects may have missed statistical significance cutoffs. Therefore, while we have generally depended on more than one corroborating piece of evidence for each connection shown, we have not required that all RNA-seq sources should score the same genes as “significantly” affected. Taken together, however, these results now provide a clearer view of the architecture of the T-cell specification gene regulatory network, showing its modular construction, and the coordination of changes in activities of its component subcircuits from stage to stage.

**Box 1: Sources of data compiled in network models**

**Table S1** presents the main data and sources used to establish specific connections in the GRN models that follow. These sources constitute RNA-seq and microarray results from studies of germline deletion of genes encoding the high mobility group factor TCF1 (encoded by *Tcf7*) (28, 76, 77), the basic helix-loop-helix E proteins E2A (*Tcf3*) and HEB (*Tcf12*) (78, 79), and the later-activated zinc finger factor associated with T-cell lineage commitment, Bcl11b (28, 80). In addition, we used studies of acute, stage-specific deletions of the ETS family subgroup member PU.1 (encoded by *Spi1*, previously called *Sfpi1* in mice) (28, 48); of Lmo2 (81); of GATA3 (28, 82, 83); of Bcl11a (28); of Erg (28); of Notch1 and Notch2 together (67); and of Runx1 and Runx3 together (51, 84). Although data for cells in the same developmental stages with complete disruption of Ikaros (*Ikzf1*) were not available, differentially expressed genes that responded to Ikaros (*Ikzf1*) zinc finger 4 deletion were also added (85). Finally, we included data from studies of acute gain of function of factors at stages after they would normally have been shut down, including PU.1 (*Spi1*) (48), and the transcription factor adaptor Lmo2 (86–88). In addition, supporting results came from studies introducing into pro-T cells acute antagonists of key transcription factors, including the natural E protein antagonist ID2 (89) or an artificially constructed dominant repressor form of PU.1 (90). Additional supporting results came from earlier perturbation studies knocking out E protein genes *Tcf3* (E2A) and *Tcf12* (HEB) or *Bcl11b* (71, 79) and studies utilizing progenitor or pro-T cell lines and acute T-cell malignancies to interrogate roles of the early-acting transcription factors Lmo2 and Hhex (81, 88, 91). For data on normal developmental expression dynamics of these genes in pro-T cells, RNA-seq and single-cell RNA-seq datasets were used (92–94), corroborated by highly curated microarray data (95). Data used were all from experiments in the mouse system, but the underlying gene expression patterns involved are largely conserved in human data (94, 96–103)(rev. in (103)). In addition to the data incorporated into **Table S1**, we consulted data from other studies for TCF1, GATA3, and Bcl11b target gene regulation as well (16, 63, 83, 90, 104, 105). Finally, note that positive and negative regulatory connections shown in the models indicate a measurable effect in the indicated developmental window, but usually not a pure Boolean function.

## 3 Overview of early thymic T-cell development

**Figure 1:**
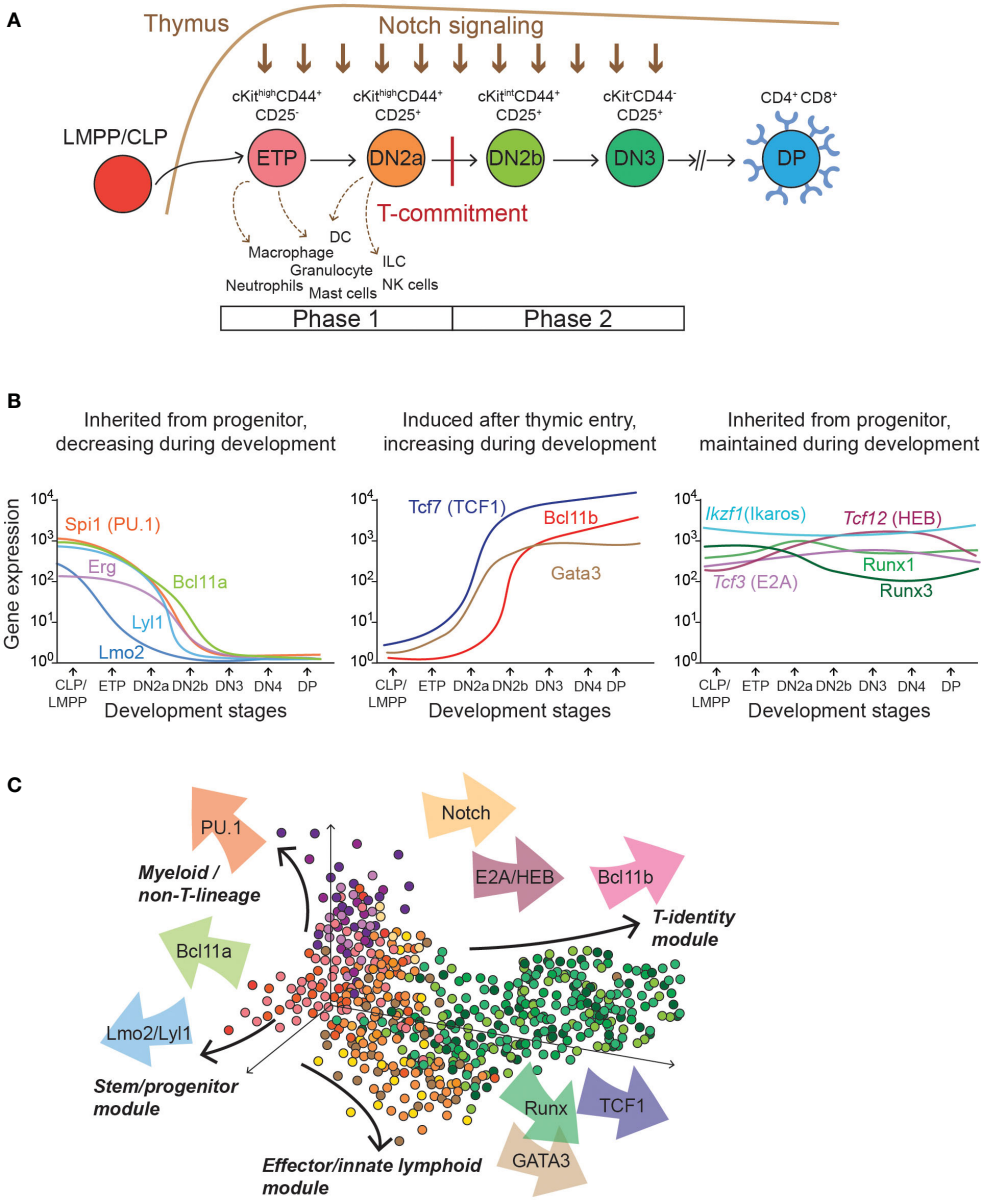
T cell development stages and transcription factor expression kinetics. **(A)** Diagram depicts different stages of early thymic T cell development that T-progenitor cells sequentially go through. Informative surface proteins that are utilized to define each stage are indicated (cKit, CD44, CD25 for ETP to DN3; CD4 and CD8 for DP). Developmental plasticity to generate alternative, non-T-lineage cells is shown with dotted arrows. Note that these alternative lineage potentials are silenced after T-lineage commitment. Lineage commitment to a T cell fate distinguishes Phase 1 (before T-lineage commitment) and Phase 2 (after T-lineage commitment). CLP, Common lymphoid progenitor, LMPP, lympho-myeloid primed progenitor. **(B)** Graphs show mRNA expression kinetics of important transcription factors involved in early T cell gene regulation programs. Left: Transcription factors inherited from the bone-marrow progenitor cells whose expressions gradually decline during T-development (*Spi1, Bcl11a, Erg, Lyl1*, and *Lmo2*). Middle: transcription factors upregulated in pro-T cells by thymic microenvironment (*Tcf7, Gata3*, and *Bcl11b*). Right: transcription factors expressed from the bone-marrow progenitor cells and stably and maintained during T cell development (*Ikzf1, Tcf3, Tcf12, Runx1*, and *Runx3*). Gene expression data was plotted by utilizing publicly available mRNA expression datasets for immune cells with curve smoothing (https://www.immgen.org) (93). **(C)** Diagram illustrates transcription factors (arrows) providing distinct forces to different gene expression program modules in individual cells.

Thymic T-development begins as hematopoietic progenitor cells possessing lymphoid potentials migrate into the thymus and interact with the cortical epithelial cells providing Notch ligands, growth factors, and cytokine signaling (106–109). This drives the developmental program shown in **Figure 1A**. At early stages, intrathymic precursor cells undergo extensive cell proliferation and upregulate some of the T-lineage associated genes. However, they still preserve multipotentiality and can differentiate into non-T lineage cells, especially if Notch signaling is withdrawn. This uncommitted stage is referred to as “Phase 1”, which includes double-negative 1 (DN1, or Early T-cell precursor, ETP) and DN2a stages. In Phase 1, both ETP and DN2a cells express high levels of cKit and CD44, but CD25 surface expression distinguishes ETP (CD25^-^) from DN2a (CD25^+^)(**Figure 1A**). Recent single-cell transcriptomics studies reveal heterogeneity in Phase 1 population both in human and mouse. The pro-T cells comprising Phase 1 actually include multiple subsets of ETPs and populations transitioning to DN2a cells that display distinct gene regulatory programs, with patterns mostly conserved between mouse and human based on single-cell and bulk analyses (94–98, 110). Of interest, the intermediate-stage ETP populations, more than the most primitive ETPs, transiently express a set of non-T lineage-associated genes (e.g. *Mpo*, encoding myeloperoxidase) even though these cells are on the T-cell pathway (94). This suggests that these genes are induced as a part of a normal developmental progression program in ETPs, and multilineage priming occurs before T-lineage commitment.

Upon sustained exposure to Notch-ligand and other thymic microenvironmental signals, progenitor cells intrinsically commit to a T cell fate, and the developmental plasticity to non-T-lineages is terminally blocked. After T-lineage commitment, pro-T cells establish a T-cell identity gene expression program and start to rearrange some forms of T cell receptor (TCR) genes. For conventional T cells, successful gene rearrangement for expression of TCRβ chain is assured by quality control at the β-selection checkpoint during DN3 stage. Other T cell precursors rearrange and express genes encoding TCRγ and TCRδ instead, to become γδ T cells. These stages from commitment to β-selection are collectively referred to as “Phase 2”, which is comprised of DN2b (cKit^int^ CD25^+^ cells) and DN3a (cKit^low^ CD25^+^ CD28^-^ CD27^low^) stages (**Figure 1A**) (111–113). Further development depends on the cells’ TCR interactions. Following β-selection, most developing T cells accumulate as CD4^+^CD8^+^ (“DP”) cells, while they complete their TCRα gene rearrangements and express for the first time the TCRαβ that they will use forever, if they are allowed to live. However, they undergo an ultra-stringent selection process to reject all cells with inadequate or highly autoreactive TCR specificity. The rare surviving cells can finally mature, undergoing divergent programs of positive selection into CD4 or CD8 single-positive cells before they emerge from the thymus, associated with “helper” or “cytotoxic” function respectively. Notably however, most core T-identity program genes that pro-T cells activate in Phase 2 are irreversibly maintained throughout all later stages of T cell development and immunological responses.

## 4 Gene regulatory network models for early T cell development through commitment

As the thymus-seeding precursor cells migrate from the bone marrow and first enter the thymus, they express transcription factors inherited from their progenitor cells in the bone marrow. Expression of many such legacy transcription factors is maintained across multiple cell divisions. While many progenitor-associated factors are turned off eventually, some legacy transcription factors maintain their expression throughout thymic T cell development, but these often adopt new roles during stage transitions by occupying different sets of genomic regions from those in hematopoietic progenitor cells. The co-existence of “inherited transcription factors” together with “newly induced regulators” in the thymic precursor cells generates numerous possible combinatorial inputs to different target genes which dynamically change their expression during developmental progression in Phase 1 and Phase 2 (**Figure 1B**). This review focuses on key driving regulatory factors and responders composing the Phase 1- and Phase 2-GRNs, in which many components show dynamic changes throughout developmental progression.

### 4.1 Gene regulatory network modules in Phase 1 and Phase 2

The newly proposed GRN that we present here highlights the observation that in each stage of early T-lineage development, subsets of target genes for a given transcription factor are often regulated in parallel ways, collectively composing a specific module that often has coherent biological function (28, 114). While the definition of a module is not precise, we use this framework to describe fairly discrete components of the developmental process which are regulated distinctly even if the cell is expressing other sets of genes at the same time. One module (defined by expression of a group of genes) may remain active consistently throughout a series of stages while other modules are sharply changing activity, or a module can be affected coherently by a perturbation that does not affect genes in other modules within the same cells. As shown below, this gives the overall process of early T cell development an “assembled” quality. Key transcription factors often appear to play roles in activity of an entire module, even while the individual target genes they act upon also have other regulatory inputs. However, it is noteworthy that a transcription factor does not define the module on its own. The same transcription factor may even work in more than one module, as shown below. Instead, different transcription factor ensembles can be seen to define distinct modules, working together to drive expression of shared target genes. They establish positive and negative feedback loops within a module, for stabilization, or between different modules, which results in dynamic gene network behavior. This modular structure is significant because the timing of transitions that individual cells make along the pathway is very asynchronous, with cells showing an ability to linger in any of several states for several cell cycles before progressing (27). The slow pace of differentiation implies that regulation of the modules that predominate in any particular stage is fairly stable; however, developmental progression depends on eventually breaking this stability. In developmental gene networks, a regulatory state is often stabilized when different transcription factors with concordant effects on the same module also support each other’s expression (115), and some examples of this are shown below. In contrast, inter-module inhibition or repression between regulators can make expression of different modules unstable and eventually incompatible.

Mutually exclusive expression of different regulators generates sharp cell-fate boundaries in binary cell fate decisions and in embryos, for example (115, 116). However, one notable feature of the T cell developmental network linkages is that the repression which is detected is often incomplete. Many repressive interactions in this system cause a dampening of expression levels but not a silencing of the target genes. This “soft” repression function enables factors with mutually antagonistic activities to coexist in the cells for days and multiple cell divisions, and it enables activities of opposing modules to overlap within an individual cell at the same time. Therefore, distinct sets of transcription factors can pull and push activities of their target modules in different directions in the same cell (**Figure 1C**), and the resultant sum of the vectorized “forces” instructs cell fate (28). In the following, we review both the regulatory circuits that maintain coherent module activity, and also the sources of antagonists that finally keep them from persisting.

The architecture of the modular subprograms within the T-cell GRN has become much clearer through recent work. The distinct subprograms that comprise the early T-cell GRNs can be categorized as 1) the T-identity module 2), the stem or progenitor module, 3) conditional access to alternative fates as a side effect of the stem/progenitor module, and 4) a cell survival and proliferation module. While the T-identity module needs to be successfully installed to provide constitutive expression of the core T-cell genes, the second module must be silenced in order to convert multipotent precursors to T-lineage committed cells. Finally, the cell survival and proliferation program in Phase 1 and Phase 2 ensures the production of sufficient number of immature T cells to accommodate later positive and negative selection. Because the T-cell identity module introduces the initiators and overall structure of the entire process, it is discussed first.

### 4.2 Installation and specification of the T-identity module in Phase 1 and Phase 2

**Figure 2:**
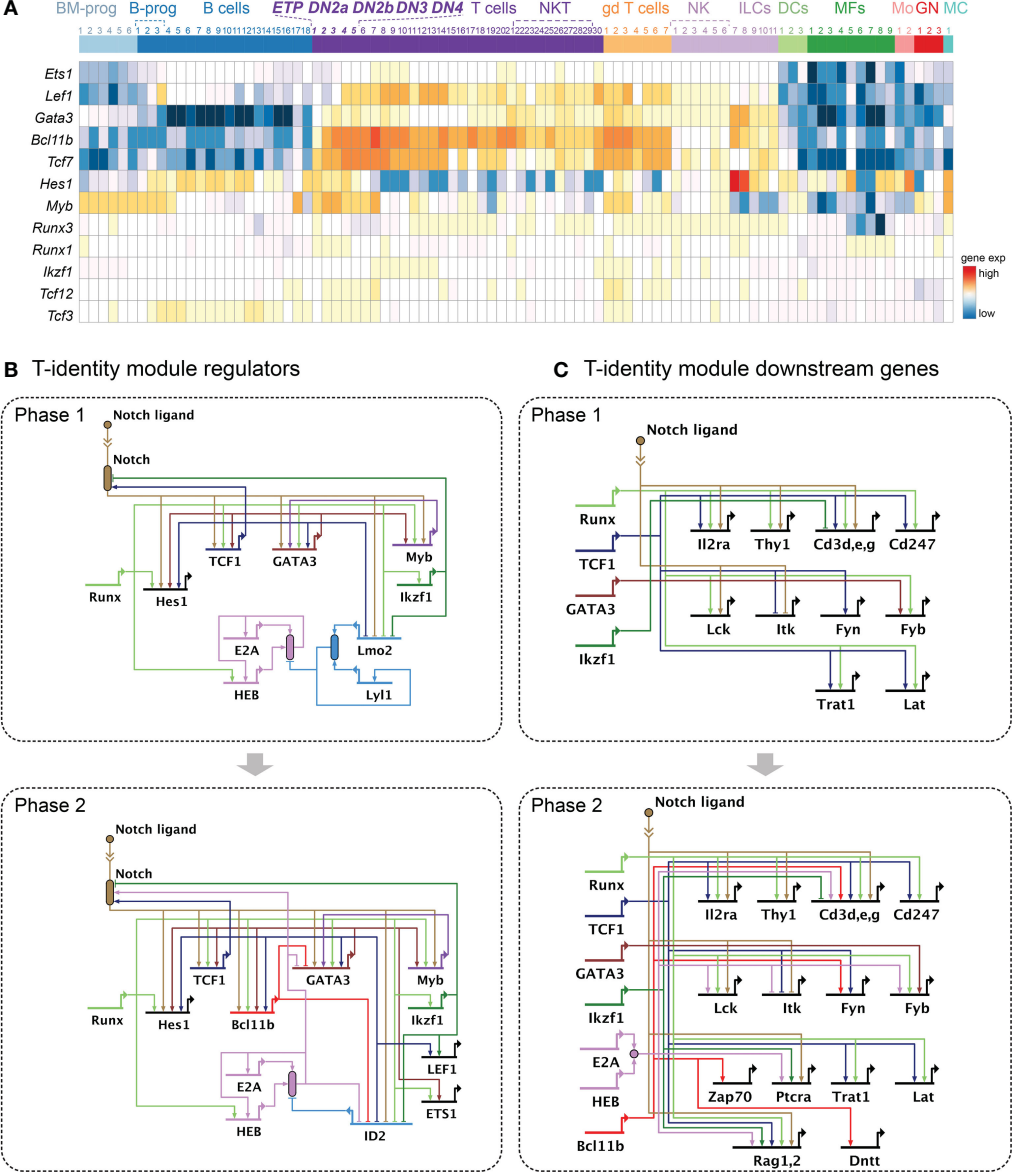
Gene regulatory networks driving the T-identity module. **(A)** Heatmap shows expression patterns of key transcription factors supporting the T-identity module across different immune cell types, using the ImmGen MyGeneSet tool (https://www.immgen.org) (93). Bone marrow progenitor (BM progen), B cells precursors (B progen), B cells, T cells, γδ T cells ("gd T cells"), natural killer cells (NK), Innate lymphoid cells (ILC)s, Dendritic cells (DC), Macrophage (MF), Monocyte (Mo), Granulocyte (GN), Mast cell (MC). See **Table S2** for the number keys. **(B, C)** BioTapestry models (71, 117) of gene regulatory network relationships described in the text. Evidence for all connections shown is in **Table S1**. Structure of BioTapestry models: Genes are shown with regulatory regions (horizontal lines) distinct from their encoded outputs (bent arrow at “promoter”). Connections with arrows represent positive regulation. Connections ending in blocking lines represent negative regulation. When gene products combine to produce a functional unit, or when activity is not a simple function of transcriptional output, a “bubble” symbol is placed to represent the emergent function from their collective activities. Both negative and positive regulation can impinge on such activity bubbles, e.g. ID factors inhibiting E protein activity without necessarily inhibiting the expression of the E protein coding genes themselves. Double chevron symbol indicates ligand-receptor interactions, here used to represent Notch ligand—Notch interaction as a source of regulatory input to other genes. Arrows consisting of dotted lines show decreasing or weak activities. These conventions are used also in **Figures 4B, C**, **5B, C**. **(B)** Current model for the T-identity module regulator transcription factors in Phase 1 (top) and Phase 2 (bottom). **(C)** Current model for the T-identity module downstream molecules in Phase 1 (top) and Phase 2 (bottom). Expression of *Il2ra* (CD25) indicates developmental progression from ETP to DN2 and DN3 stages in mice. Surface markers: *Thy1*, *Cd3* clusters genes (*Cd3d, Cd3e, Cd3g*), Cd247; TCR signaling molecules: *Lck, Itk, Fyn, Fyn, Trat1, Lat, Zap70, Ptcra*; TCR rearrangement molecules: *Rag1, Rag2, Dntt*.

Commitment to the T-cell fate involves two major events: 1) terminal blockade of alternative lineages, concomitant with 2) acquisition of T-identity gene expression. The gene network instructing the constitutive expression of the T-lineage defining genes will be referred to as the T-identity module. Successful establishment of the T-identity module is pivotal because the core T-identity established in early thymic developmental phases is robustly maintained even after these progenitor cells become mature T cells, long after they leave the Notch ligand-rich thymic environment. The “T-cell markers” include CD3 clusters and TCR signaling mediators, as well as transcription factors necessary for the induction and maintenance of these marker genes. The T-identity module requires activities of Notch signaling, TCF1, GATA3, Runx transcription factors, and Ikaros starting from Phase 1, then receives critical positive inputs from the E protein complex and Bcl11b as pro-T cells progress through Phase 2. Expression patterns of the key factors in different immune cell populations are shown in **Figure 2A** (data from www.immgen.org (93)).

#### 4.2.1 Notch signaling as an indispensable driver

Notch signaling is an absolute requirement for initiation and establishment of the T-identity module. In the absence of Notch signaling, B cells develop in the thymus instead of T cells, whereas constitutive expression of intracellular domain of the Notch (ICN) in the bone-marrow progenitor cells induce extrathymic T cell development (118–120). In addition, many if not all thymic seeding progenitors need to be primed by some level of Notch signaling in the bone marrow. Whereas Jagged-class Notch ligands expressed in the bone marrow do not signal as strongly as the Delta-class Notch ligands in the thymic microenvironment (121–123), this prior experience seems to be important for the cells to acquire competence to initiate the T cell program (124) A thymic seeding progenitor population that had received Notch signaling before thymic entry also exists in humans, supporting a physiological contribution of Notch signaling to T-lineage specification from the prethymic stages in both humans and mice (97). The stromal cells in the thymic cortex express the strongest ligands for signaling through Notch1, mainly Delta-like 4 (Dll4), as well as Dll1 and to a lesser extent Jagged 2 (Jag2). These engage with Notch receptors (Notch 1-Notch3) on the thymic precursor cells with varied affinities (122, 125). This ligand-receptor interaction induces proteolytic cleavages in the Notch receptor, releasing the ICN to the cytoplasm. Then, the Notch ICN interacts with DNA-binding transcription factor RBPJκ and functions as co-activator, recruiting other transcriptional coactivators and chromatin modifying enzymes (122, 126).

Although Notch1 is most strongly expressed throughout, acute deletion of different Notch family genes in pro-T cells shows that Notch1 and Notch2 cooperate to induce the T-identity module in Phase 1 cells by activating genes encoding essential transcription factors (e.g., *Tcf7*, *Myb*, *Gata3*, *Hes1*), the core-T cell marker genes (*Cd3g*, *Cd3e*, *Thy1*) and the useful DN2-DN3 stage marker *Il2ra* (in mouse; not in human at this stage). Later, in Phase 2, Notch signaling induces genes necessary for TCR rearrangement and signaling (*Rag1*, *Rag2*, *Lck*, *Ptcra*) *(* 67)(**Figures 2B, C**). Notch actions in the T-cell identity module are not hit-and-run; the signals must be sustained through lineage commitment and then to sustain most cells’ viability into the beginning of β-selection [rev. by (127)]. Notably, however, the target genes regulated by Notch signals either positively or negatively change markedly between Phase 1, early Phase 2, and the end of Phase 2 (67). The shifting but essential roles of Notch signaling in pro-T cells provide an example of context-dependent shifts in regulator deployment which are seen for other factors as well (15, 49, 51).

#### 4.2.2 Notch-induced effectors TCF1 and GATA3, cooperative but nonredundant

Notch-activated targets in Phase 1 cells include genes encoding TCF1 (*Tcf7*) and GATA3, which are pivotal for instituting the T-identity module (**Figures 2A, B** top). The activation of *Tcf7* by Notch signaling appears to be direct, although its maintenance becomes Notch-independent in Phase 2 (67, 76). The functional importance of TCF1 and GATA3 in T-lineage specification is demonstrated by transgenic animal models that lack *Tcf7* or *Gata3* expression. *Tcf7-* or *Gata3*-deficiency in pro-T cells abrogates T-cell development from the earliest Phase 1 stage (76, 77, 82, 104, 128–130). *Tcf7* deletion in bone-marrow derived progenitors using *Vav1-*Cre caused developmental arrest at ETP stage and allowed abnormal transcriptome clusters to accumulate among thymocytes in steady state, based on single-cell RNA-seq (scRNA-seq) (63). In accord with these results, another recent single-cell transcriptome study using dual guide-RNAs (gRNAs) to disrupt *Tcf7* or *Gata3* specifically in Phase 1 cells showed that the precursor cells lacking TCF1 completely failed to enter the normal T-cell developmental trajectory, while those lacking GATA3 failed to progress properly (28). While *Tcf7* and *Gata3* both depend on inputs from Notch signaling and Runx family transcription factors to be turned on, they also create a possible stabilization circuit for early T-cell specification by positively regulating each other as well (28, 77, 82)(**Figure 2B**).

Despite these positive feedbacks, acute *Tcf7* deletion results in different impacts on the developing pro-T cell population than acute *Gata3* deletion in the same Phase 1 developmental time window, based on scRNA-seq data. TCF1 and GATA3 regulate distinct target genes, indicating that these factors do not perform redundant functions. Also, studies using gain-of-function approaches suggest that TCF1 overexpression is completely different from the effect of high dosage GATA3 in pro-T cells. A high level of TCF1 in the mouse bone-marrow progenitor cells can upregulate essential genes in the T-identity module, such as *Gata3* and *Bcl11b*, even without Notch signaling, causing cells apparently to bypass the ETP stage (77). In contrast, elevated GATA3 levels block T cell development, promoting deviation to an alternative, mast-cell lineage in the absence of Notch signaling, and killing pro-T cells if Notch signaling is sustained (79, 82, 83, 131). This difference in part involves the different impacts these factors have on genes within proliferation and survival modules used in Phase 1 cells, including effects on *Kit*, *Il7r* and *Spi1* (see below).

In pro-T cells, TCF1 and GATA3 instruct T-cell development by upregulating many T-program genes (*Notch1, Notch3, Hes1*, *Gata3*, *Bcl11b*, *Lef1, Ets2*, *Il2ra*, all *Cd3* genes, *Cd247*, *Tcrb*, *Lat, Fyn, Rag1, Rag2*, and *Trat1* by TCF1; *Myb, Ets1, Tcf7*, and *Bcl11b* by GATA3)(**Figure 2C**). Importantly, both TCF1 and GATA3 are required together for the initial induction of Bcl11b, which will be important in Phase 2, suggesting that multiple transcription factor inputs are non-redundantly required (16).

**Figure 3:**
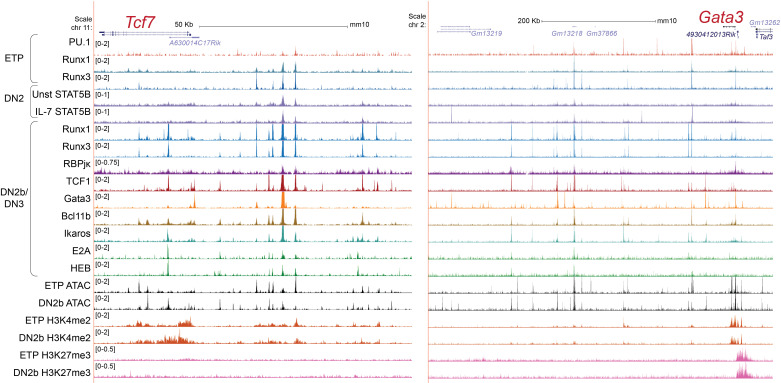
Transcription factors and chromatin state markers in Tcf7 and Gata3 enhancer regions. UCSC genome browser tracks show transcription factor occupancy, histone markers, and chromatin accessibility profiles near the *Tcf7* locus (left) and *Gata3* locus (right) in the indicated stage (67, 84, 93, 101, 133, 134). H3K4me2 represents active enhancers and H3K27me3 marks repressive chromatin regions. Unst STAT5B: unstimulated STAT5B, IL-7 STAT5B: IL-7 stimulated STAT5B.

As TCF1 and GATA3 are necessary to initiate the T-lineage specification program, the positive regulatory factors inducing these transcription factors are also critical. TCF1 and GATA3 positively regulate each other and Runx transcription factors also provide supportive inputs, as described below. The critical regulatory elements of the *Tcf7* gene in T-lineage cells, 30-40kb upstream of its promoter regions (132), are also occupied by RBPJκ, Runx factors, GATA3, and TCF1, consistent with direct positive regulation by these factors (**Figure 3** left, in "DN2b/DN3" samples; note that both *Tcf7* and *Gata3* in this figure are transcribed from right to left). Similarly, an enhancer region 280 kb downstream of the *Gata3* gene, which is known to be necessary for *Gata3* expression in the T-lineage (54), is occupied by RBPJκ, Runx factors, TCF1, Bcl11b, and GATA3 by Phase 2 (**Figure 3** right, “[i.e., "DN2b/DN3"] samples).

The strong force that TCF1 exerts to drive the T-cell program involves reprogramming of chromatin accessibility and long-range looping. TCF1 overexpression in fibroblasts opens the chromatin regions near the T lineage-associated genes, which are naturally demarcated by repressive histone marks in fibroblasts (135). Recent studies demonstrate a potent role of TCF1 in chromatin architecture remodeling in pro-T cells, later DP thymocytes, and mature T cells (136, 137). In Phase 2, TCF1 occupies key sites in evolutionary conserved topologically associating domains (TAD), i.e. regions of chromatin containing clusters of regulatory elements that interact within the TAD but are usually insulated from other TADs. When TCF1 binds to the inter-TAD sites along with CTCF (a transcription factor that can anchor chromatin architecture), this co-occupancy weakens insulation of the TAD boundaries and enables intermingling of TADs, potentially allowing new enhancer-promoter interactions. In addition, TCF1 establishes long-range looping around the T cell genes and marks the surrounding regions with H3K27ac (136).

#### 4.2.3 Multitasking positive roles of Runx family factors

Runx transcription factors are broadly expressed in all hematopoietic lineage cells, but they exert context-specific functions by switching their interaction sites across the genome. The expression of Runx1 and Runx3 is established prior to the thymic entry, and these two paralogs are co-expressed within individual Phase 1 and Phase 2 pro-T cells alike (51, 93, 94). The sum of Runx1 and Runx3 activities, measured by total binding by ChIP-seq, is maintained stably throughout the early T-lineage developmental process, suggesting that overall Runx availability between Phase 1 and Phase 2 does not dynamically change (51). However, the DNA binding profiles of Runx factors from hematopoietic stem cells (HSCs), ETP, DN3, DP, naïve CD4 T cells, and regulatory T cells (Tregs) demonstrate that a large fraction of the Runx binding sites displays highly cell type-specific occupancy (84). The sites occupied by Runx factors in ETPs are almost as different from those in DN3 cells as the sites occupied in completely different hematopoietic lineages, explaining the fact that Runx factors positively or negatively regulate substantially different sets of target genes in Phase 1 pro-T cells than in Phase 2 pro-T cells (51). Thus, the gene network role of Runx factors within the T-identity module changes with increased developmental progression, like the role of Notch signaling.

Due to functional redundancy between Runx1 and Runx3 in the pro-T cell stages, their roles in early T-developmental stages were previously under-detected when only one of the paralogs was disturbed. However, upon disruption of CBFβ, the common co-factor of all Runx factors, almost all stages of pro-T cells disappear, demonstrating the importance of Runx factors in early stages of thymic T-development, and NK cell development as well (138). Accordingly, acute deletion of Runx1 and Runx3 together in Phase 1 or Phase 2 pro-T cells results in a severe developmental block and dysregulation of developmentally important genes. The Runx target genes defined by stage-specific loss-of-function and/or gain-of-function reveal the striking positive influence of Runx1 and Runx3 on regulating the core T-cell identity genes, which include *Tcf7*, *Gata3, Hes1, Bcl11b, Myb, Ikzf1, Tcf12, Lef1*, and *Ets1* (transcription factors); *Cd3g, Cd3d*, *Cd3e*, *Cd247*, and *Thy1* (T cell surface markers); *Lck*, *Lat, Fyb, Trat1*, and *Rag2* (TCR signaling and rearrangement molecules) (51)(**Figures 2B, C**).

Of interest, cell-type-preferential Runx binding sites show distinct, stage-specific enrichment profiles for motifs of other factors, including PU.1, E2A, ETS, or TCF/HMG factors, suggesting that Runx factors may be cooperating with distinct partners to be recruited to these different binding sites in a cell-type specific manner (84). Indeed, Runx factors are identified as “popular” functional collaborators of other factors in Phase 1 and Phase 2, as they work with PU.1 in Phase 1, and at least with GATA3 and Bcl11b in Phase 2 (48, 71, 133). As Notch/RBPJκ binding sites are frequently co-enriched with Runx motifs (67), and there is substantial overlap between Notch and Runx-regulated target genes (**Figure 2**), it is likely that Runx interacts functionally at some sites with Notch/RBPJκ as well. Runx interacting co-factors can indeed alter Runx binding site choices. PU.1 binding accompanies Runx binding at a major fraction of its Phase 1 sites. If added to Phase 2 cells, where Runx binds different sites, PU.1 recruits Runx1 to its binding loci even though these sites possess lower quality Runx motif sequences than the starting Phase 2 sites (133). This PU.1-mediated Runx redistribution actually depletes Runx occupancy from the higher quality Runx binding sites, but this can be rescued by increasing the Runx availability levels (84). Notably, introducing additional Runx1 to Phase 1 pro-T cells results in precocious Runx DNA binding to post-commitment specific sites, which hastens activation of some Phase 2 targets of Runx factors and accompanies substantial acceleration in developmental progression. These results show that multiple co-factors may compete for the limited amount of Runx factors, and that this competition has a strong impact on the Runx DNA binding site choices as well as T-development speed.

#### 4.2.4 Ikaros family factors: A universal lymphoid requirement

A zinc finger protein, Ikaros is encoded by *Ikzf1* and highly expressed in lymphoid progenitor cells. Thymic progenitor cells maintain a stable level of *Ikzf1* expression throughout all stages, and Ikaros is necessary for normal T-development as well as B and innate lymphoid cell development (139–142). An impaired form of T cell development survives a complete *Ikzf1* null mutation (143), but this is likely due to redundancy with its relative, *Ikzf2*, which is also expressed in early T-cell precursors (144, 145). Disruption of zinc finger 4 of *Ikzf1* affects DNA binding, resulting in different developmental defects, which range from DN2/DN3 cellularity loss, abnormal β-selection, and delayed progression, to T-cell lymphoma (85, 142). Notably, Ikaros target genes are highly context dependent, and distinct sets of genes were uniquely regulated in Phase 1 vs. Phase 2, and beyond. The positive targets affected by the zinc finger 4 deletion at least include *Lef1, Spib, Ptcra, Cd8a, Cd8b1, Rag1* and *Rag2*, while its negative targets include *Cd4*, all relevant both to Phase 2 (**Figures 2B, C** lower panels) and to the orderly transition of the cells succeeding in β-selection to later stages. Other evidence has also indicated that Ikaros can act as a quantitative damper on Notch signaling in this transition (146, 147). Some Notch-induced genes antagonized by Ikaros acquire H3K27me3 histone marks in DN3 stage by Ikaros-mediated Polycomb repressive complex 2 recruitments to these regions (134). However, the importance of *Ikzf1* for prethymic lymphoid precursor development (148) makes it important to revisit the targets and gene regulation mechanisms of Ikaros by stage-specific perturbation specifically in the earlier T-lineage context as well.

#### 4.2.5 Basic helix-loop-helix E proteins, E2A and HEB

E proteins are class I basic helix-loop-helix (bHLH) family members and among them, E2A (encoded by *Tcf3*), E2-2 (encoded by *Tcf4*), and HEB (encoded by *Tcf12*) contribute to early thymic T cell development (78, 149–153). As E proteins form homodimers or heterodimers with other bHLH or HLH family member proteins, their activity is regulated by the expression of the other heterodimerization partners as well as of the E protein coding genes themselves. For instance, ID-family transcription factors harboring an HLH domain but not a basic region specifically antagonize the DNA-binding activity of E proteins (154). Also, under pre-leukemic conditions, Lmo1, Lmo2, Lyl1, and SCL are suggested to sequester E protein activity in thymic progenitor cells (155–157). In pro-T cells, E2A/HEB activity sharply increases between DN2a and DN3, as measured by target gene effects (**Figures 2B, C**, compare upper vs lower panels), even though E2A itself is relatively unchanging across Phase 1 and Phase 2, and HEB level only increases by about fourfold (78, 93). Since there is no detectable decrease in ID protein expression across this interval, it is possible that before this transition, stem/progenitor-associated partners like Lyl1 or SCL were previously sequestering these E proteins into complexes with alternative activities (see below).

The E2A/HEB heterodimer is essential for activating many genes involved in the T-identity module, especially as the cells move into Phase 2 (*Notch1 Notch3, Tcf3, Tcf12, Cd3d, Cd3e, Fyb, Ptcra, Lck, Rag1, Rag2*). E2A and HEB directly bind to enhancer regions near *Notch1, Notch3*, *Rag1*, and *Rag2*, and these regions require E proteins to increase chromatin accessibility during the Phase 1 to Phase 2 transition (78). A recent study shows that E2A activates expression of the *Rag1* and *Rag2* recombinase genes through distinct enhancer regions in pro-T cells, transforming the spatial organization of chromatin around the *Rag1-Rag2* locus (158). E protein activity subsequently enforces proliferation arrest and TCR gene rearrangement quality control at the β-selection checkpoint (159).

#### 4.2.6 Bcl11b and the Phase 1–Phase 2 transition

The onset of Bcl11b expression in thymic pro-T cells precisely marks commitment to the T cell fate (16). This is a relatively late event which is tightly linked to the Phase 1-Phase 2 transition (**Figures 2B, C**, lower). Bcl11b-deficient animals reveal that many genes crucial for T cell identity are Bcl11b-dependent (*Cd3g, Cd3d, Cd3e, Lat, Ptcra, Zap70, Notch3, Hes1, Dntt*) (80). Consistent with these results, Bcl11b deficient pro-T cells fail to assemble TCRβ and cannot progress beyond Phase 2 due to a defect in establishing the T-cell program (71, 80, 160). Many genes likely to be direct activation targets of Bcl11b (possessing Bcl11b binding near their putative regulatory domain) are also sensitive to the deletion of Runx1, indicating that this partner factor may also participate in Bcl11b-mediated positive gene regulation (80).

*Bcl11b* not only requires multiple positive inputs for its expression (TCF1, GATA3, Notch, and Runx) but also needs to undergo a major chromatin change (161), involving a compartment flip of at least 1 megabase (162) and removal of Polycomb repression marks (163), in order to be activated. This makes its advent later than might be predicted from gene network considerations alone. Like germline *Bcl11b* knockouts (164–166), disruption of the *Bcl11b* locus at pre-commitment stage (using *Vav1*-Cre or retroviral Cre) or immediately post-commitment (using proximal *Lck*-Cre) results in impaired developmental progression from DN2 to DN3 stage (80, 167).

The impact of Bcl11b has a notable overlap with the effects of E proteins. However, their overlapping target genes require inputs from both Bcl11b and E proteins, as E proteins and Bcl11b do not directly regulate each other nor bind frequently to linked sites. Bcl11b does protect E protein activity indirectly by repressing *Id2*, but this effect may only account for a select minority of the overlapping gene regulation effects (80). Thus, the T-identity module is initiated from Phase 1 by activities of Notch, TCF1, GATA3, Ikaros, and Runx factors, and further specified and strengthened by E protein factors and Bcl11b in Phase 2.

Entry into the T-cell program also requires additional factors which still require better characterization to determine the genes they regulate in early pro-T cells specifically. These other critical regulators include the bifunctional transcription factor Myb (168–170), the zinc finger repressor Gfi1 (171, 172), and ETS (E twenty-six) family transcription factors, which bind a motif that is among the most highly enriched in the active chromatin regions in all early pro-T cell subsets (51). Although single knockouts of ETS factors have not given strong phenotypes in pro-T cells, a large number of ETS family members with near-identical DNA binding specificities are expressed in overlapping patterns in these cells (173, 174), indicating that their function is probably reinforced by redundancy. Future work is needed to place these factors in the T-identity gene network module, and to elucidate how the T-identity module is robustly maintained later throughout all subsets of conventional T cells.

### 4.3 The stem or progenitor module

**Figure 4:**
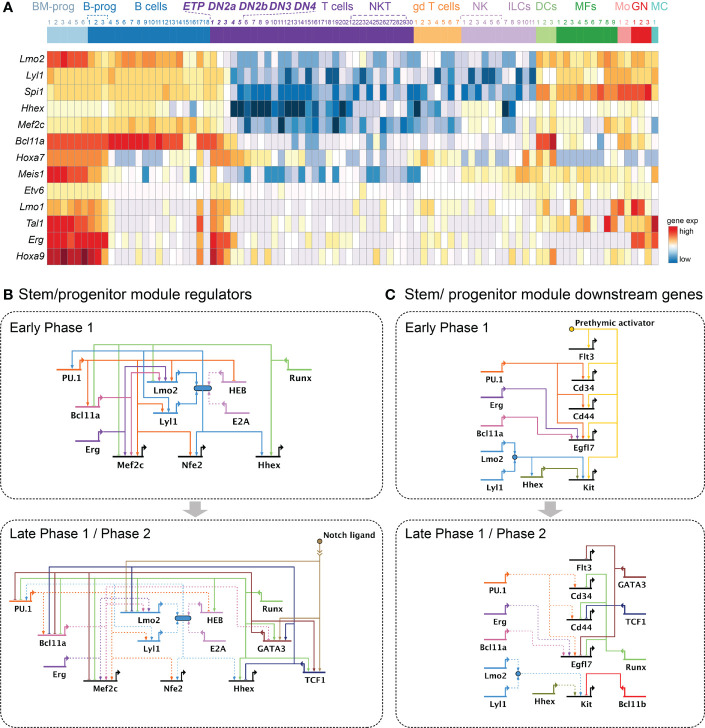
Gene regulatory networks controlling the stem or progenitor module. **(A)** Heatmap illustrates expression patterns of important transcription factors involved in the stem or progenitor module. Heatmap includes the same immune cell populations as **Figure 2A**. **(B, C).** Gene regulatory network models as in **Figure 2**. **(B)** Current model for key transcription factors shaping the stem or progenitor module in Phase 1 (top) and Phase 2 (bottom). **(C)** Current model for the stem or progenitor module downstream molecules in Phase 1 (top) and Phase 2 (bottom). Growth factor receptors: *Flt3, Kit*; Growth factor molecule: *Egfl7*; ETP marker genes: *Cd34, Cd44*.

The T-cell identity program is not established on a blank slate, but rather intrudes on a previously established progenitor-associated gene regulation program when the cells enter the strong Notch signaling environment of the thymus. Many transcription factors handed down from hematopoietic stem cells to pro-T cells contribute to the progenitor program in Phase 1, then they are repressed after T-lineage commitment. A failure of silencing the stem/progenitor module regulators in the later T-developmental stages is often linked to various types of leukemia as well as failure of developmental progression (175). This group of transcription factors includes PU.1, Lmo2, Lyl1, Hhex, Bcl11a, Erg, Mef2c, Meis1, Hoxa9, and possibly also Etv6, Mycn, and Hopx (**Figure 4A**), and these factors regulate both common and unique sets of genes (28, 48, 90–92, 175–183).

#### 4.3.1 PU.1: A positive driver sustaining the stem or progenitor state

The best-studied legacy regulator involved in the progenitor module is an ETS family transcription factor, PU.1, encoded by *Spi1.* PU.1 also acts in a pioneer factor-like way to set much of the chromatin accessibility landscape in Phase 1 cells (48). This mode of action is similar to its mechanism of function in myeloid cells (184), although deployed to somewhat different sites. The expression of PU.1 is highest in ETP, then gradually declines as pro-T cells transition to Phase 2. PU.1 provides strong and broad positive inputs to a large number of other progenitor-associated transcription factor coding genes (*Lmo2, Lyl1, Bcl11a*, and *Mef2c*)(**Figure 4B**) and genes encoding progenitor cell-related surface molecules (*Cd34, Cd44*, and *Egfl7*)(**Figure 4C**). The DNA binding profile of PU.1 in hematopoietic stem and progenitor cells (HSPC) vs. thymic precursor cells (ETP, DN2a, and DN2b cells) shows that at least 50% of the PU.1 DNA binding sites in HSPCs remain occupied by PU.1 in ETPs, which may provide continuity with PU.1’s function supporting the stem/progenitor program in pro-T cells (84).

Many of the genes functionally regulated by PU.1 in Phase 1 pro-T cells encode molecules used in myeloid cells as well, including receptors for myeloid-cell promoting cytokines (185). In hematopoiesis, the importance of PU.1 for myeloid lineage and dendritic cell fates is well-known, where it establishes chromatin accessibility and induces lineage specifying gene expression through collaborations with C/EBPα and IRF4/8 (15, 186–192). However, strong Notch signaling in the thymus restricts the full actions of PU.1, ensuring that pro-T cells do not actively follow the myeloid developmental path (89, 193, 194). In part, Notch signaling acts to silence expression of the partner PU.1 uses for several myeloid programs, C/EBPα, *via* the Notch-activated repressor Hes1 (105). Thus, in the context of strong Notch signaling, even moderately elevated levels of PU.1 can be tolerated within early T-cell precursors (89).

PU.1 maintains the stem and progenitor state both by direct positive regulation of targets in the same module, and by inhibition, direct or indirect, of the activity of factors promoting progression to T-cell commitment. The single-cell perturbation analysis study previously cited found that while PU.1 did not affect TCF1 or GATA3, it reduced the expression of later-acting progression-associated regulators, including HEB (*Tcf12*) (28) (**Figure 4B**), with strong impacts on their predicted target genes as identified by the SCENIC algorithm (39). Thus, without diverting T lineage development, PU.1 could act as a brake on the speed of differentiation along the T-cell pathway.

#### 4.3.2 Lmo2, Lyl1 and the connection of stem-ness to leukemia

Other critical regulators of the stem or progenitor module are Lmo2 and the bHLH factor Lyl1. These form a complex with E protein (e.g. E2A or HEB), a GATA family factor, and an Ldb1 “bridge”, in which the Lyl1/E protein heterodimer interacts with DNA and the association of Lmo2 with Lyl1 stabilizes the complex (155, 156, 195, 196). In hematopoietic stem and progenitor cells, the complex often involves the related bHLH factor SCL instead of Lyl1 (197, 198). Lyl1 (or SCL) heterodimerization with an E protein like E2A or HEB confers an altered DNA binding specificity, such that Lyl1/E2A or SCL/E2A dimers bind different genomic sites than E protein/E protein dimers (156). Of note, the Lyl1/E2A complex in prethymic precursors appears to be important to support lymphoid programs and T cell potential, and to generate true lymphomyeloid primed progenitors (LMPPs) expressing genes shared with ETPs, such as *Flt3*, *Dntt*, *Bcl11a*, and even *Notch1* itself (199). During early T cell development, Lyl1 is important to make ETP to DN2a progression possible and to turn on *Gfi1* (200), which encodes a zinc finger repressor protein that is crucial for survival of early lymphoid progenitors in the presence of Notch signals (172). Recent studies also show a supportive role of Lmo2 in maintaining the T-lineage competence of immortalized progenitor-like cells (81, 195). Thus, although silenced in mature T cells, Lmo2 and Lyl1 contribute to the T-lineage developmental competence of hematopoietic progenitors.

However, overexpression of Lmo2 or Lyl1 in thymocytes does not drive the T cell program. Instead, it causes T-cell lymphoma/leukemia, and when Lmo2 is involved, it causes increased expression of genes associated with progenitor fates (86–88, 201–203)(**Figures 4B, C**, top). Under these conditions, the Lmo2/Lyl1 protein complex directly activates *Hhex*, which upregulates *Kit* expression (encoding the growth factor receptor cKit) and promotes self-renewal (91) at the expense of T-lineage differentiation. The Lyl1/Lmo2 complex can also upregulate *Spi1* (encoding PU.1) itself (88). Such gain of function phenotypes suggest that Lyl1 and Lmo2 could be regulating the same genes in normal Phase 1 pro-T cells, although the definitive tests still need to be done. Of note, *Lmo2* expression normally declines in ETPs much earlier than expression of *Lyl1*. However, PU.1 provides positive input to both, in a positive feedback circuit (**Figure 4B**, top): acute disruption of *Spi1* in Phase 1 pro-T cells downregulates both *Lmo2* and *Lyl1* expression, while ectopic expression of *Spi1* in early Phase 2 increases *Lmo2* expression (48, 89). This suggests that PU.1 at high levels can re-activate *Lmo2* for a limited time even after T-lineage commitment.

#### 4.3.3 Distinct developmental roles for viability-critical factors Bcl11a and Erg

Acute CRISPR/Cas9-mediated target gene disruption in Phase 1 pro-T cells has demonstrated previously unknown roles of *Bcl11a* and *Erg* (28), both factors that have been difficult to study in T-lineage cells with conventional knockouts because of their essential roles in hematopoietic progenitor cell survival. Bcl11a is a zinc finger factor that has expression closely associated with lymphoid potential in progenitors, in B lineage cells, dendritic cells (especially pDC), and Phase 1 pro-T cells, then parallels PU.1 (*Spi1*) in its downregulation in T lineage cells. Thus, its expression pattern in pro-T cells is the opposite of that of its relative Bcl11b. Germline *Bcl11a* integrity is known to be essential for lymphoid development, but has mainly been implicated in survival for lymphoid cells (92, 180). Erg, an ETS factor similar to Fli1, is expressed specifically in stem and progenitor cells, granulocytes, mast cells, and pro-T cells through Phase 1, shutting off later than PU.1 and Bcl11a in Phase 2 [data for both from (93)]. At the single-cell transcriptome level, Bcl11a and Erg both contribute to the stem and progenitor module (28, 92, 94).

The recent data from single-cell transcriptome analysis shows that acute loss of *Bcl11a* in ETPs accelerates T lineage developmental progression along a largely physiological path. The loss of Bcl11a causes downregulation of progenitor-associated genes (*Mef2c, Lmo2, Hoxa7*, and *Egfl7*) together with upregulation of the T-lineage promoting regulatory gene *Gata3* (28). While *Bcl11a* knockout effects parallel those of *Spi1* (PU.1) knockouts, they are not identical, indicating distinct if overlapping sets of targets. In contrast to Bcl11a, the proto-oncogene Erg has not been implicated in any positive role specific to lymphocyte development before, as it is a sharply dose-sensitive regulator that is indispensable for survival of hematopoietic stem cells and for the embryo (181, 204). With acute Cas9-mediated deletion and in the presence of anti-apoptotic Bcl2, however, a striking effect of *Erg* knockout can be seen specifically in Phase 1 pro-T cells. First, expression of other stem/progenitor signature genes (*Mef2c, Lmo1, Lmo2, Egfl7*, and *Meis1*) is markedly decreased in the Erg-knockout cells. Second, the loss of Erg most prominently shifts the entire developmental trajectory of the Phase 1 pro-T cells to a novel path of development, one in which normally-unused genes *Klf4, Id1*, and others increase expression aberrantly (28). Thus, in contrast to Bcl11a and PU.1, Erg does not only sustain the stem/progenitor program but also blocks a particular cryptic, alternative pathway that was not known to be available before in T-cell developmental conditions.

#### 4.3.4 The unknown “prethymic activator” and additional contributors

The stem/progenitor module regulators in Phase 1 thymocytes very likely are preserved from the particular minority of multipotent progenitors that achieve success in colonizing the thymus, and so it is likely that some of these factors contribute positively to this very early event. As noted above, progenitor factors PU.1, Bcl11a, and Lyl1 are crucial for the generation of bone marrow cells competent to go to the thymus, and the Lyl1 partner Lmo2 is similarly essential to preserve T cell developmental potential in immortalized progenitor cells. However, it is not clear exactly which of their direct gene regulatory targets are the decisive ones for priming the ability to enter the thymus, thus separating T cell precursors from most ILC precursors. More inputs are also needed to explain the expression patterns of *Flt3, Cd34, Cd44, Egfl7*, and possibly *Kit*. Thus, a crucial early potentiating mechanism for T-cell development remains undefined. 

Other factors expressed during the Phase 1 period could also contribute important regulatory inputs to targets in the stem/progenitor module, including Mef2c, Meis1, Hoxa9, Etv6, Hhex, and Mycn. These are all implicated in T-ALL (reviewed in (205)), similarly to Lyl1 and Lmo2 (86–88, 91), but are not yet adequately explored in the normal pro-T cell context. Hoxa9 is implicated in generating lymphoid-competent multipotent precursors (206) and has been reported to synergize with Runx1 to drive induced pluripotent cells towards a T cell pathway (207). Hhex is also required for generation of lymphoid-competent cells and has been suggested to play roles in sustaining a T-lineage competent state (208, 209), although it delays T-lineage developmental progression by stimulating extra self-renewal (91). Mef2c, also robustly expressed in early ETPs (94), may actively oppose the T cell program. Like PU.1 at high levels, Mef2c is reported to block T cell development by antagonizing Notch signaling (182), although it remains to be seen whether this is also true of Mef2c at natural levels in nonmalignant cells. One problem with determining the roles of these factors in normal pro-T cells has been technical: as the roles of these factors are confined to early stages, it has been difficult to disrupt or neutralize them within the T-lineage differentiation context faster than they are downregulated naturally. Thus, while the stem/progenitor module presented here already includes mutual activation and feed-forward network circuit motifs (PU.1 and Lmo2/Lyl1; PU.1 and Bcl11a on common targets) that could produce metastable persistence, it is likely that other module participants could further reinforce this system property.

#### 4.3.4 Turning off the stem/progenitor module

The repressive forces antagonizing the stem or progenitor module also begin in Phase 1, and they fully suppress this program in Phase 2. This antagonism is mediated by a distinct set of T identity-associated regulators, primarily Runx transcription factors (Runx1 and Runx3), TCF1, and GATA3 **(** **Figure 4**, lower panels). Although PU.1 utilizes Runx transcription factors as its predominant binding partners, Runx factor activities most commonly oppose the actions of PU.1 on individual shared functional target genes (48, 51, 133). Runx transcription factors are also potent at counteracting the stem/progenitor module by repressing the majority of key transcription factor coding genes supporting this program (*Mef2c, Lmo2, Lyl1, Bcl11a, Tal1*, and *Meis1*) in a highly concentration-sensitive way (84). As the cells pass from Phase 1 to Phase 2, Runx1 begins to collaborate with GATA3 to repress *Spi1* (PU.1) (51, 210) and turns off the downstream target genes of PU.1 as well, *Cd34* and *Cd44* (51).

TCF1 and GATA3 are not only indispensable for the T-identity program but also a source of discrete inhibitory inputs to the stem or progenitor module. Intrathymic Notch signaling activates TCF1 and GATA3 early in the ETP stage (76, 77, 82, 128, 132, 135, 211, 212), and they are co-expressed with genes associated with the progenitor module in individual ETPs and DN2a cells in mouse (94). Acute deletion of *Tcf7* causes pro-T cells to stay behind at a progenitor-like state with sustained high expression of the genes associated with the stem/progenitor module (*Cd34, Mef2c, Bcl11a, Lmo2*, and *Hhex*), instead of progressing toward the T-lineage commitment stage. The contribution of GATA3 to this module is partially mediated by its repression of *Bcl11a* within the Phase 1 cells (28)(**Figure 4B**, lower). In addition, it plays a seemingly direct role in silencing *Spi1* during T-lineage commitment. A recent study shows that in mice, GATA3 and Runx1 together interact with a specific regulatory element in intron 2 of *Spi1* (PU.1) to cause and maintain PU.1 repression in Phase 2 (210). Although a different mechanism is apparently used in humans (213), forced expression of GATA3 in pro-T cells silences *Spi1* prematurely (and blocks access to myeloid developmental fates) in mouse and human cells alike (83, 102, 104). Moreover, GATA3 inhibits an additional set of progenitor genes (*Hopx, Hoxa9*, and *Flt3*), all associated with self-renewal (28), which are not strongly repressed by Runx factors (**Figures 4B, C**, lower).

Together, the activity of the stem and progenitor module in early period of Phase 1 is maintained by the integrative efforts from PU.1, Lmo2 and Lyl1 complex, Bcl11a, and Erg, and potentially also Hhex, Etv6, Meis1, and Hoxa9. However, Notch signaling and Runx factors start to offset this activity from early ETP stage. As Notch and Runx factors activate TCF1 and GATA3, these factors provide additive force against the progenitor module. As a result, the major positive regulators in this module are downregulated with *Lmo2* first, followed by *Hhex*, *Mef2c*, *Bcl11a*, and then *Spi1*, *Lyl1*, and last *Erg*. This process pushes the Phase 1 precursor cells finally out of the progenitor state as they develop.

### 4.4 The effector and innate lymphoid modules in Phase 1 and Phase 2

**Figure 5:**
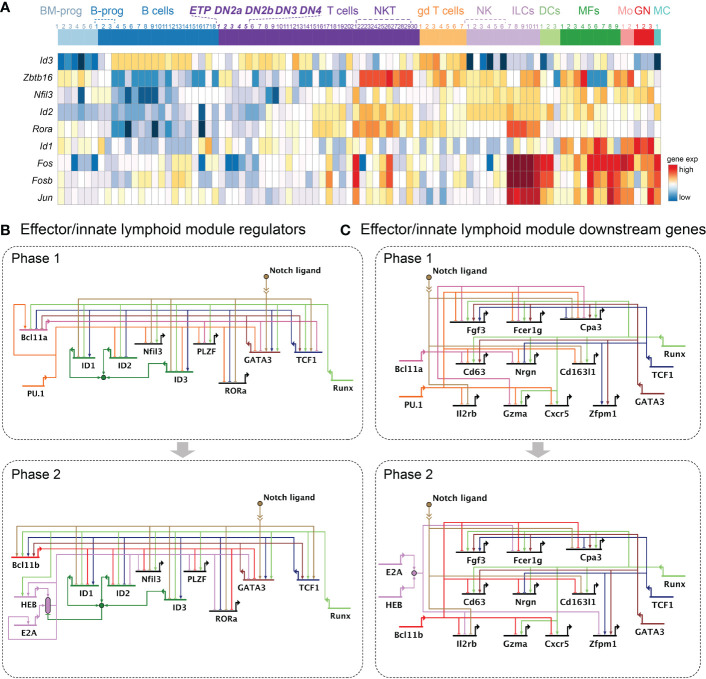
Gene regulatory networks regulating the effector or innate lymphoid module. **(A)** Heatmap represents expression patterns of transcription factors supporting the effector or innate lymphoid module. Heatmap shows the same immune cell populations displayed in **Figure 2A**. **(B, C).** Gene regulatory network models as in **Figure 2**. **(B)** Current model for transcription factors regulating the effector or innate lymphoid module in Phase 1 (top) and Phase 2 (bottom). **(C)** Current model for the effector or innate lymphoid module downstream molecules in Phase 1 (top) and Phase 2 (bottom). Growth factor, immunoglobulin, scavenger, or chemokine receptors (*Fgf3, Fcer1g, Cd163l1, Il2rb, Cxcr5*), enzymes (*Cpa, Gzma*), and signal transduction associated molecules (*Cd63, Nrgn*) involved in immune cell effector function and innate lymphoid cells are shown. Not all genes discussed in the text are shown.

Developmental potential for the B cell and myeloid cell fates depends on factors exogenous to the T cell program, like EBF1 and Pax5 for the B cell program, C/EBP factors for the neutrophil and macrophage programs, and IRF family factors for macrophage and dendritic cell programs. The B-cell option becomes unavailable relatively quickly for T-cell cohorts that have access to it at all (214), potentially associated with the early loss of Mef2c (182), and the myeloid-related options are less prominent by the time pro-T cells reach the DN2a stage. Instead, the NK and ILC lineage potentials become more preferred alternative lineage choices in DN2a cells, especially in the absence of Notch signaling (16, 94). The gene network components supporting the NK and ILC fates will be referred to as “the effector and innate lymphoid module” here. Notably, the effector/innate lymphoid module is often fueled by the same transcription factors necessary for the T-identity module, such as Runx factors, TCF1, and GATA3, whose expression is maintained in all T-lineage cells. The distinctive transcription factors separating the effector and innate lymphoid module from the T lineage include ID family proteins (mostly *Id2*), PLZF (encoded by *Zbtb16*), NFIL3, RORα, and Fos/Jun family members (*Fos, Fosb, Jun*) *(* 215–225). Although some of these can be activated by acute stimuli, these genes are generally not highly expressed in ETPs, with a few exceptions (**Figure 5A**).

#### 4.4.1 Crossover roles for T-lineage factors in an “effector or innate lymphoid” module

Runx1 and Runx3 generally antagonize the stem/progenitor module in ETPs and generally favor T-lineage progression, but they also globally activate another distinctive program, including a suite of regulatory genes associated with effector and innate lymphoid cells plus downstream genes associated with innate lymphoid cells, γδ T cells, and other effectors. In fact, Runx factors, especially Runx3, are indispensable for the normal development and functions of ILCs (218, 226–228). A modest increase of Runx1 levels in Phase 1 pro-T cells quickly upregulates the innate lymphoid cell-associated regulators *Zbtb16* and *Nfil3.* In addition, effector genes associated with innate-lymphoid cells (*Fcer1g, Nrgn)*, γδ T cells (*Cd163l1*), and mast cells (*Cpa3, Cd63*), and the rarely-expressed *Fgf3*, are upregulated by Runx1 overexpression and inhibited by *Runx1/Runx3* double knockout (84). Although this gene set does not mimic markers of a known mature cell type, its highly coherent regulation, described in this section, suggests a specific alternative developmental program. The association with NK or ILC programming, based on *Zbtb16* and *Nfil3*, is supported by the developmental behavior of Runx-overexpressing cells (84). Consistently, if Notch signaling is withdrawn, Runx1 overexpression in ETPs causes preferential diversion to the NK-associated lineage at the expense of the myeloid lineage choice. However, Runx factors do inhibit all ID proteins (*Id1, Id2, Id3*) as long as Notch signaling is present, keeping the full innate program conditionally on hold. Hence, the activity of Runx proteins in Phase 1 guides pro-T cells out of the progenitor state and blocks the myeloid program, while driving cells towards lymphoid fates by upregulating both T- and ILC-associated genes (84).

Although GATA3 and TCF1 are essential for generating functional T cells, recent studies have shown their roles in ILC development as well (132, 229–233). GATA3 is necessary for development of all helper-like ILC subsets and their functions (232–234), and ILCs may express much higher levels of *Gata3* than pro-T cells (**Figure 2A**). The early ILC progenitor (EILP) population is also dependent on TCF1 upregulation, in which TCF1 provides positive inputs to key ILC genes while blocking DC development from EILP (229, 235). During early thymic T-development, GATA3 and TCF1 positively function in the effector and innate lymphoid module by activating the gene encoding the GATA cofactor *Zfpm1* (by TCF1 and GATA3)(**Figure 5B**, top), and also activating non-T effector molecules *Cpa3, Cd63*, and *Fcer1g* (by GATA3), and *Fgf3* (by TCF1 and GATA3)(**Figure 5C**, top; **Table S1**). Of interest, TCF1-regulated genes in EILPs often overlap with TCF1 targets in pro-T cells, suggesting that it generally promotes a T or innate lymphoid program. Many of the non-T targets are inhibited by Bcl11b in pro-T cells in Phase 2 (**Table S1**, also see below). The physiological relevance of this pathway is shown by the fact that normal ETP-DN2a pro-T cells within the mouse thymus transiently express all of these genes (in a “wave”) before commitment to the T-cell pathway (94). This suggests that TCF1 may provide some extent of common inputs to the effector and ILC program in both EILPs and Phase 1 pro-T cells.

#### 4.4.2 A relay of repression mechanisms between Phase 1 and Phase 2

If these genes truly provide access to ILC potential, then they should be silenced to ensure lineage specification to the T cell fate. First, Notch signaling widely represses the genes associated with the innate lymphoid/effector module (*Id1, Id3, Nfil3, Rora, Il2rb*, and *Cpa3, Cd163l*) (**Figures 5B,C**, lower panels). In addition, this module gets repressive inputs from two distinct, seemingly opposite groups of transcription factors. The first group, consisting of PU.1 and Bcl11a, inhibits the effector and common lymphoid program in Phase 1, and then hands over this job to the second group of transcription factors in Phase 2, which are E protein complexes and Bcl11b.

PU.1 shows strikingly powerful opposition to the effector and innate lymphoid module by inhibiting ID proteins, Jun family members, as well as other ILC-associated transcription factors (*Zbtb16, Id2, Id3, Nfil3, Jun, Jund, Rora*, and *Pou2af1*). In addition, multiple downstream molecules in this module (*Fcer1g, Fgf3, Gzma, Gzmb, Il2rb, Cd63, Nrgn, Cxcr5, Cd163l1*, and *Cpa3*) are negatively regulated by PU.1 (28, 48). Similar to PU.1, but to a weaker extent, Bcl11a also functions to inhibit expression of the lymphoid/effector module (*Zbtb16, Gzma, Gzmb, Cd63, Zfp105, Fgf3, Cpa3, Fcer1g*, and *Nrgn*). Notably, Bcl11a and GATA3 inhibit each other (28). This suggests that PU.1 and Bcl11a are not generally permissive to all of the non-T lineage potentials in Phase 1, but in fact provide a critical counterbalance to keep pro-T cells from deviating to the ILC-lineage when excessive activity of Runx factors, GATA3, and TCF1 pushes the cell state towards the effector like-lymphoid lineage.

As pro-T cells progress to DN2 stage, PU.1 and Bcl11a expression declines, and the role of repressing the effector and innate lymphoid module devolves upon E protein complexes and Bcl11b. The E2A/HEB complex provides strong opposing force to the effector and innate lymphoid module by inhibiting expression of key ILC and innate-like lymphoid transcription factors (*Zbtb16, Id2, Nfil3, Ikzf2, Sox5, Junb*, and *Rora*) as well as by damping down expression of *Gata3* (78, 79). In addition, E protein activity is important for Notch1 expression, which is also necessary for preventing ILC potential from being expressed (78, 236). These E protein effects would all be countered by ID2, explaining in part why ID2, despite its inability to bind DNA, is so important for NK and ILC development. *Tcf3/Tcf12* (E2A and HEB) double-deficient ETPs display an abnormal chromatin accessibility signature, which mimics the ATAC-profile of ILC2-precursor cells in the bone marrow (78). In accordance with this, disruption of E protein function by deleting *Tcf3* and/or *Tcf12* or by overexpressing their antagonistic factor ID proteins results in abnormal ILC- and NK-like cell development in the thymus (78, 89, 236–238). These results reaffirm that the E2A/HEB complex is necessary to block developmental access to the innate lymphoid-like functionality.

However, later in T cell development, the balance of E proteins to ID proteins is repeatedly tipped whenever the TCR complex is stimulated (239–241). Thus, E protein activity levels alone cannot guarantee that the cells will stay within the T cell lineage long-term. Another indispensable transcription factor repressing the innate lymphoid/effector module is thus Bcl11b. The onset of Bcl11b expression in early thymic T-development closely coincides with T-lineage commitment, and Bcl11b plays pivotal roles afterward for establishing T-cell identity (16, 28, 80, 165, 166). The most abundant Bcl11b interaction co-factors include multiple repressor complexes, and, during T lineage commitment, Bcl11b broadly represses the NK and innate lymphoid cell programs (80, 166, 167). This is achieved by blocking expression of multiple regulators (*Zbtb16, Id1, Id2, Nfil3, Rora, Zfp105*, and *Pou2af1*) as well as downstream genes (*Gzma, Cpa3, Il12rb, Fgf3, Cd163l1, Nrgn, Fcer1g, Cxcr5, Cd63*, and *Il2rb)* in the effector and innate lymphoid modules (80).

A recent single-cell transcriptome analysis highlights that Bcl11b-deficient pro-T cells begin to swerve out of the conventional αβ T cell pathway with *Id2* derepression and abnormal activation of AP-1 factor-associated genes (28). This leads to a cascade of regulatory changes, shown in part in **Table S1** by separate comparisons between WT and Bcl11b-knockout cells in specific single-cell clusters: the slight initial differences detected as the paths first separate, within cluster 5; and the widening divergences mid-cascade in the comparisons between clusters 2 and 0. At the end of this cascade in the Bcl11b-knockout cells, further upregulation of *Id2* culminates with a shutoff of Notch signaling and upregulation of *Rora*, as the cells leave the T cell pathway (28). Consistent with this dysregulated transcriptional program, *Bcl11b* deletion results in abnormal lineage-conversion to an NK-like population (called induced T-to-natural killer, ITNK cells) in the thymus, which express genes associated with conventional NK cells and show cytolytic activity to tumor cells and even to OP9 stromal cells (166, 167). Combinatorial deletion of *Bcl11b* and *Id2* or *Bcl11b* and *Zbtb16* shows that Bcl11b-mediated negative regulation on these transcription factors is necessary to block alternative lineage choices to NK- and ILC-like fates (80).

In summary, the effector and innate lymphoid module represents a battery of genes that are remarkably consistently regulated by the activities of factors that cut across their usual progenitor vs. T-identity roles. These genes are activated by three of the same inputs that drive the T-identity module, i.e., Runx factors and the Notch-induced factors GATA3 and TCF1, but they are restrained from expression normally by three distinct sets of antagonists: 1) the progenitor factors PU.1 and Bcl11a, 2) the climax T lineage commitment factors Bcl11b and E proteins, and 3) environmental Notch signaling itself. The Notch, GATA3, and TCF1 inputs form a classic “incoherent feed forward” motif (242) as the inducer and the factors it induces exert opposite effects on this module. The silencing of these genes by progenitor factors early switching over to Bcl11b and E proteins later clearly leaves a window of vulnerability, in which these genes may be activated transiently, and indeed this is when the genes in this module show a burst of activity in normal late-Phase 1 cells (precommitment DN2a cells). To the extent that these genes indeed indicate competence to switch to a different developmental branch than normal αβ T cells, this discontinuity in repression provides a window of opportunity.

### 4.5 Cell survival and proliferation module

The thymus generates about 50 million immature T cells per day, and then outputs 1-2 million mature T cells after selection per day in the young adult mouse, although only a few hematopoietic progenitor cells enter the thymus every day (243, 244). This yield is supported by remarkable cell expansion before various checkpoints and selections. Hence, the program supporting cell proliferation and survival is a critical component of Phase 1 and Phase 2 GRNs.

#### 4.5.1 Cytokine receptor-driven survival and proliferation

In Phase 1, growth factors Flt3-ligand and cKit-ligand are necessary to yield optimal number of output pro-T cells (110, 245–248). The expression of Flt3 receptor is limited to the least mature ETP population, whereas cKit is expressed in a range of cells from ETP to DN2a (cKit^high^)/DN2b(cKit^intermediate^) stages until T-lineage committed cells eventually downregulate it in Phase 2 (94, 110, 249). Among two forms of cKit-ligand or stem cell factor (SCF), membrane bound and secreted forms, the membrane-bound form of SCF is necessary for sustaining the Phase 1 population size. The SCF in thymic endothelial cells is particularly important for the ETP population, whereas membrane-bound SCF in thymic epithelial cortex cells is more important for DN2 cells than ETPs (250). However, the exact molecular mediators linking Flt3 and cKit signaling to the proliferation/survival transcriptional programs remain unclear.

In late Phase 1 and Phase 2, IL-7 signaling plays an essential role in cell survival and proliferation. The exact time windows affected could be complex. Fate-mapping studies tracing the origin of thymic T cells suggest that all T cells are derived from precursors expressing IL-7 receptor (IL-7Rα) at some level; in steady state, mice deficient in IL-7 or IL-7Rα manifest severe defects in thymic cellularity due to cell cycle arrest and high apoptosis rates (251–255), with a relative emptying of the pro-T cell niches (256). Some thymus-seeding cells in humans as well as mice indeed come from IL-7Rα^+^ prethymic precursors (termed Common Lymphoid Precursors or IL-7R^+^ LMPPs) (97, 257–260), especially in fetal thymus (261, 262). However, the great majority of ETPs in postnatal mice do not appear to express this cytokine receptor at RNA level (93, 94). Instead, the expression of IL-7Rα in the thymic precursor cells sharply increases at the late stage of Phase 1 (ETP to Bcl11b^-^ DN2a transition stage), peaks at around T-lineage commitment (Bcl11b^+^ DN2a and DN2b stage), and then gradually decreases as pro-T cells develop through DN3 and DN4 stages (263–265). During the DN2a/2b phases, *Il7r* expression overlaps with *Kit* expression. The dynamic kinetics of IL-7R expression coordinated with the developmental progression may provide the maximum efficiency for the cell expansion during the time that it is most needed.

In the case of IL-7R signaling, the main mediators are known. The Jak3-STAT5 pathway and Jak3-NFAT2 pathways are directly activated by IL-7 signaling, and both upregulate anti-apoptotic genes *Bcl2* and *Bcl2l1* (266, 267). Consequently, ectopic expression of *Bcl2* or deletion of *Bim* (proapoptotic molecule) provides partial rescue for the severe cellularity loss in IL-7 signaling-deficient mice (266–268). STAT5 target genes specific to the pro-T cell compartment were defined in a recent study by using Cas9/CRISPR to knock out both *Stat5a* and *Stat5b* in pro-T cells expressing a *Bcl2* transgene to minimize the survival defect in STAT5-knockout cells (269). In this system, both uncommitted and newly committed pro-T cells could be compared from control and double-knockout populations. Knocking out *Stat5a* and *Stat5b* simultaneously resulted in dysregulation of genes associated with cell cycle, cytokine signaling feedbacks, and the mTOR/metabolic pathway. Surprisingly, however, these knockouts showed minimal changes in key transcription factors specifying T-cell identity, either before or after commitment. This suggests that the cell survival/proliferation module in Phase 2 is not necessarily intimately linked to the cell-identity specifying modules, in contrast to results in peripheral T cells (270). Thus, cytokine responses *via* STAT5 do not appear to instruct pro-T cell state changes *via* lineage-specifying transcription factor changes.

#### 4.5.2 Survival and proliferation effects of lineage-determining transcription factors

Nevertheless, certain lineage specifying transcription factors also exert population effects in Phase 1 and/or Phase 2. The effects of Phase 1 and Phase 2 transcription factors on well-established growth control genes in these cells are summarized in **Table 1**, based on data in **Table S1**. Notch signaling, PU.1, Lmo2/Lyl1 complex, TCF1, and GATA3 are not only involved in the cell identity-specifying modules, but are also essential for regulating the size of the Phase 1 pool. Notch signaling induces proliferation of thymic progenitor cells both in humans and mice (271–273). Notch activation target genes include *Myc*, which is potent at driving cellular proliferation (67). Also, gene set enrichment analysis suggests that loss of Notch signaling enriches expression of genes associated with apoptotic programmed cell death pathways (67).

**Table 1:** Inputs to growth control genes from developmental network regulators.

Growth control-related gene	TFs activating	TFs repressing
*Il7r*	?	Bcl11b, Ikaros, Lmo2, STAT5a/STAT5b
*Jak2*	PU.1	E2A
*Jak3*	?	?
*Kit*	Lmo2	Bcl11b
Myc family		
* Bmyc*	Bcl11b, PU.1	Runx, TCF1
* Myc*	Notch, E2A	Runx
* Mycn*	E2A, Lmo2, Runx	Bcl11a
Stat5a*	E2A, GATA3, Lmo2	?
*Stat5b*	E2A, Lmo2	?

Relationships shown are compiled from **Table S1**. TFs activating: loss of function of the indicated transcription factors causes the growth control gene expression level to go down. TFs repressing: loss of function of the indicated transcription factors causes the growth control gene expression level to go up.?, denotes that the regulators were not identified because the indicated gene did not meet the statistical criteria threshold as differentially regulated by any of the perturbations studied in **Table S1**. Note: Bcl2 is not listed as a target gene because a Bcl2 transgene was present in many of the studies in **Table S1**.

*Stat5a RNA levels are also reduced in Stat5a, Stat5b double knockout samples, but it is not clear whether this is an autoregulatory transcriptional effect or due to nonsense-mediated decay.

In Phase 1, acute deletion of PU.1 can result in a ~5-10-fold reduction in Phase 1 cell number from the *in vitro* culture system, which cannot be fully restored by forced expression of anti-apoptotic molecule Bcl-xL (90). PU.1 activates genes associated with cell cycle progression (*Ccnd1* and *Cdk18*) and supports expansion of both ETP and DN2 cells, suggesting that PU.1 is still needed for optimal cell proliferation even after some genes in T-identity modules are upregulated in DN2 stage (48, 90, 133). Another important early Phase 1 transcription factor involved in cellular proliferation is Lmo2. Lmo2 interacts with DNA replication complexes to regulate the DNA synthesis rate in hematopoietic progenitor cells, and it increases the pool of self-renewing population in Phase 1 in a gain-of-function setting (86, 274, 275). Also, Lmo2 upregulates Bcl2 expression in pro-T cells, which contributes to maintain the size of the Phase 1 cell pool (81), consistent with the effect of the Lmo2 partner Lyl1 on the ETP-DN2a pool size *in vivo* (200). Consistently, constitutive expression of Lmo2 in bone marrow progenitor cells results in higher frequencies of ETPs in cell cycle upon bone marrow chimera reconstitution, which leads to T-ALL due to the accumulated number of ETP-like cells in the thymus (275).

TCF1 is critically important for supporting survival and proliferation of T lineage-competent cells starting from Phase 1. In the absence of *Tcf7*, the overall thymic DN cellularity is severely impaired and thymic progenitor cells display reduced frequency of cells in cell cycle (276, 277). In addition, *Tcf7*-deficient ETPs downregulate genes associated with DNA damage response and show increased cell death; constitutive expression of survival factor Bcl-xL does not rescue the early developmental defect resulting from *Tcf7*-deficiency (76, 77). This effect is strong enough to raise the concern that even when supported with a *Bcl2* transgene, many cells in the pro-T population that survive *Tcf7* knockout could be irrelevant bystander cells if the true T-precursors were annihilated. Another key transcription factor positively contributing to the proliferation/survival module is GATA3 (82, 128, 278). Acute knockdown of GATA3 expression using shRNA causes severe loss of the ETP population, ascribed to a cell intrinsic defect in proliferative capacity. Of note, transgenic expression of anti-apoptotic factor Bcl2 can rescue ETP survival in these cases, but not sufficiently to restore normal T cell development (82).

In contrast, the recent study deleting *Erg* using CRISPR-Cas9 has highlighted a further unexpected role of Erg in regulating cell proliferation in pre-commitment stage (28). The loss of *Erg* in Phase 1 not only pushes the cells to exit faster from the ETP state, but also results in a large burst of progeny with upregulated Myc family activity (28), as assessed by the SCENIC algorithm (39). This suggests that Erg may function normally as a “brake” for the proliferation program in Phase 1.

At the later stage of Phase 2, pro-T cells need to slow down cellular proliferation to prepare for TCRβ assembly, which involves cleavage and rearrangement of DNA strands in the G1 cell-cycle phase. The E protein complex provides antagonizing forces against the cell survival and proliferation module near the end of Phase 2. E2A/HEB double knockout mice and E2A/LAT double knockout mice (transgenic mice unable to signal through TCRβ) display hypercellularity in the thymus, because of aberrant proliferation of DN3 cells which cannot maintain cell cycle arrest (159, 279). Hence, E protein-deficient mice are highly susceptible to T-cell lymphoma (149, 280).

In summary, cell cycle and survival controls greatly shape the output populations of the T-cell specification gene regulatory network, and the network regulators indeed influence cell cycle state directly. As shown in **Table 1**, the positive and negative regulatory inputs to these growth control genes extend across the dichotomy between progenitor-associated and T-specification factors, emphasizing the importance of modulating expression of this gene set correctly. However, the main environmental signals controlling proliferation *via* IL-7R and STAT5 apparently do not directly affect the network architecture itself.

## 5 Constraints on network state changes by three-dimensional (3D) chromatin configuration and chromatin states

In classic gene network analysis, it is assumed that any sites with binding motifs for specific factors can be bound any time that those factors are expressed. However, this is not necessarily true in post-embryonic mammalian developmental systems, where chromatin configurations can pose a constraint. Examples from reprogramming to pluripotency have clearly shown that a concerted, multi-factor, stepwise process is needed to alter chromatin states to make many transcription factor binding sites accessible (281–283). In the T cell specification gene regulatory network, at least one important gene reveals how strong the accessibility constraint can be, separate from the availability of trans-acting factors, to affect the response of a target gene to its regulators. The *Bcl11b* gene requires positive inputs from four known factors, TCF1, Runx1, GATA3, and Notch, but all four of these inputs are available already in ETP stage, whereas *Bcl11b* is not activated normally until late DN2a stage, which is days later in cells from young adult mice. When the two different alleles of *Bcl11b* were tagged with distinct fluorescent reporter proteins in the same cell nucleus, longitudinal live imaging and flow cytometry showed that the two alleles do not necessarily get activated at the same time. Instead, in individual cells from the same mouse, the two *Bcl11b* alleles were activated in a non-coordinated, stochastic manner in which activation of the second allele could be delayed by for 1-4 day(s) after the first (161). This suggests that even though all *trans*-acting transcription factors may be fully capable to turn on one allele, slow *cis*-acting mechanisms affecting the chromatin state, such as removal of repressive histone marks and accumulation of *cis*-acting non-long coding RNA (10, 163), also need to operate properly on each allele separately before the gene responds. Such mechanisms could greatly affect the dynamics of network state switching even without affecting the topology of the regulatory relationships.

The mechanisms involved affect chromatin at two size scales. The mammalian genome has two different types of long-range interaction regions at large scales, the “A” and the “B” compartments, which correspond roughly to active and silent chromatin respectively (284, 285). Within a compartment, chromatin folds into topologically associated domains (TADs), in which DNA interactions such as enhancer-promoter looping preferentially occur within the same TAD (286, 287). During T-lineage commitment, pro-T cells reorganize compartments as well as the TADs within them (162). The genomic regions manifesting compartment reprogramming represent only a small fraction, but when this “compartment flip” occurs, it is near developmentally important genes. For instance, the major enhancer regions about ~850kb downstream of *Bcl11b* show a dramatic 3D compartment flipping as the gene is activated: these regions lie in an inactive compartment (compartment B) in HSPC, ETP, and DN2a stages, but the compartment status sharply changes to active (compartment A) across the region as *Bcl11b* turns on (162). Consistently, this genomic region also physically migrates from the nuclear lamina at the progenitor stage to the nuclear interior as pro-T cells reach the DN3 stage (10). Similarly, particular genomic regions near Phase 1-specific genes *Meis1, Cd34, Mef2c*, and *Bcl11a* display active-to-inactive compartment flip (A-to-B), while enhancer regions of *Ets1* and *Gata3* gradually convert from the inactive to the active compartment (B-to-A) as these genes are accessed by new enhancers.

Although not directly tested, these concerted large scale chromatin 3D architecture changes may create barriers for some transcription factors. For instance, Runx transcription factors can choose different stage-specific DNA binding sites across the genome as noted before (51), and interestingly, local chromatin accessibilities do not serve as major barriers. However, at a larger scale, Runx factor binding is almost excluded from the inactive B compartment, and even when Runx levels are experimentally raised, Runx1 still does not occupy genomic regions within the inactive compartments (84). It remains to be elucidated by which mechanism pro-T cells reprogram the compartment-level genome organization, and how this shapes transcription factor activities and gene expression program.

At a smaller scale, for accurate gene regulation, pro-T cells undergo dynamic, concerted changes in intra-TAD interactions, chromatin accessibilities (determined by ATAC-seq or DNase-seq), and re-establishment of histone marks (92, 93, 162). These changes are particularly pronounced during T-lineage commitment, i.e. the DN2-to-DN3 transition, suggesting that epigenetic marking is coordinated to drive cell state conversion (52, 93, 162). Chromatin accessibility in Phase 1 is largely governed by PU.1, which can bind closed chromatin sites and open them, then keep its binding sites from closing (48). As PU.1 expression declines when pro-T cells progress from ETP to DN2b stages, many PU.1 binding sites lose chromatin accessibility; this supports a pivotal role of PU.1 in maintaining the Phase 1-preferential open chromatin regions (48). By contrast, many of the long-range loops near the T-identity genes appear to be dependent on other factors, which include TCF1, Bcl11b, and E2A (136, 158, 162). Thus, the epigenetic dynamics modulating gene network action are affected by both current *trans*-acting transcription factor activity and baseline *cis*-regulatory chromatin states.

## 6 Concluding remarks

To convert progenitor cells possessing multilineage developmental potential to T-lineage committed cells, each cell needs to silence the stem/progenitor module and block activation of the effector/innate lymphoid module, while launching the T-identity module. These modules of coherently co-regulated genes are valuable to recognize as distinct units of developmental programming, because in various situations an effect on a population survival module, for example, can be completely distinct from an effect on a differentiation module per se, even if both could alter the output of the process. Noting that correct development requires both activation of a differentiation module and repression of a progenitor state module, potentially through separate mechanisms, can be valuable to shed light on perturbed development as seen in leukemias and lymphomas. As discussed above, different combinations of transcription factor activities generate distinct forces on each gene regulatory module, which in turn determines the state of a given cell. While the network models shown here are most likely lacking some key components that have not yet been closely studied, their structure already helps to bring several principles into focus.

Transcription factor cooperation within a module can be stabilized by positive feedback loops. For instance, TCF1 with GATA3, and GATA3 with Myb provide cycles of activating connections to each other, which support the T-identity. Similarly, PU.1 and Lmo2 positively regulate each other, and this positive feedback loop needs to be disrupted by receiving inputs from other transcription factors to silence the stem/progenitor module, in order for T-lineage development to progress. The relative stability of relationships within a module can provide insight into why cells appear to undergo multiple cell cycles within each substage in Phase 1 before moving to the next (74, 94, 243).

Notably, mutually repressive connections are also seen in this system, and they often result in inter-module antagonism. For example, Bcl11a and GATA3 repress each other, which generates antagonism between the stem/progenitor module vs. the T-identity module. In addition, E2A/HEB complex and ID2 inhibit each other, which is a critical determinant of gene expression programs favoring the T-identity module vs. the effector/innate lymphoid module. However, direct mutual repression is far less prevalent in these T-cell specification network circuits than would be expected from classic GATA-PU.1 opposition models of cell fate decision (116). In more frequent cases, activity of a factor like PU.1 inhibits expression of a T-identity gene like *Tcf12* (HEB), while GATA3 clearly represses *Spi1* in a dose-dependent way, in one-way antagonistic relationships. 

The opposition between different modules is also driven by indirect circuits regulating target genes. Although Notch signaling does not regulate expression of PU.1 itself, Notch signaling broadly inhibits PU.1-activated target genes; whereas PU.1 downregulates Notch-induced genes (67, 89). Interestingly, Runx-inhibited target genes also show striking overlap with the genes positively regulated by PU.1, which is surprising, considering that PU.1 physically interacts with Runx factors to co-bind to PU.1 target genes (51, 133). Thus, collaborative binding does not necessarily imply concordant functional impacts. At DN2b stage, morever, Runx1 and GATA3 cooperatively repress PU.1 itself. Therefore, Notch signaling and Runx factors together oppose PU.1 activities, which generates antagonisms between the T-identity vs. the stem/progenitor module.

Notch signaling and Runx factors show mosaic patterns of cooperation. Notch and Runx factors commonly activate the genes necessary for the T-identity module and co-occupy shared genomic regions in Phase 2. However, Notch signaling opposes Runx activities supporting the effector and innate lymphoid module. Similarly, Notch signaling works in an incoherent feed-forward circuit to repress the effector and innate lymphoid module genes that are being activated by the Notch-activated factors TCF1 and GATA3. Thus, rather than working in a linear pathway, transcription factors act combinatorially to distinguish different programs.

The effector and innate lymphoid module is actively suppressed by different cohorts of transcription factors throughout Phase 1 and Phase 2. In Phase 1, PU.1, Bcl11a, and Notch signaling collectively inhibits this module, but then E2A/HEB complex and Bcl11b take over this task as Notch signaling continues in Phase 2. It is important to note that E2A/HEB activity is regulated by competitive complex formation. In Phase 1, Lmo2 and Lyl1 must partner with E protein, which may sequester the E2A to bind alternative genomic sites. In addition, ID proteins (mainly ID2) expressed at any stage are also capable of trapping bHLH factors into non-DNA binding complexes. Thus, liberating E2A/HEB heterodimer activity by inhibiting their competitors is critical for suppressing the effector and innate lymphoid module and supporting the T-identity module.

Together, the gene regulatory network modules and transcription factors shaping each module activities reviewed here highlight how transcription factors collaborate to initiate, stabilize, synergize, oppose, or silence different gene expression programs. These intra-module and inter-module dynamics suggest how T-cell fate specification is instructed by gene regulatory network architecture.

## Author contributions

BS compiled the data from published sources, generated the network models, and wrote the paper. ER advised on sources, edited the network models and wrote the paper. Both authors planned the project before writing and edited the manuscript before submission. All authors contributed to the article and approved the submitted version.

## Supplementary material

Supplementary Table 1 Comprehensive datasets for genes affected by transcription factor perturbations, with Accession Numbers of sources, and with transcription factor binding data where available.

Supplementary Table 2 Heatmap keys describing different immune cell populations shown in in **Figures 2**, **4**, **5**.
